# The genetic background shapes the susceptibility to mitochondrial dysfunction and NASH progression

**DOI:** 10.1084/jem.20221738

**Published:** 2023-02-14

**Authors:** Giorgia Benegiamo, Giacomo V.G. von Alvensleben, Sandra Rodríguez-López, Ludger J.E. Goeminne, Alexis M. Bachmann, Jean-David Morel, Ellen Broeckx, Jing Ying Ma, Vinicius Carreira, Sameh A. Youssef, Nabil Azhar, Dermot F. Reilly, Katharine D’Aquino, Shannon Mullican, Maroun Bou-Sleiman, Johan Auwerx

**Affiliations:** 1 Laboratory of Integrative Systems Physiology, École polytechnique fédérale de Lausanne, Lausanne, Switzerland; 2 Janssen Research and Development, LLC, Raritan, NJ, USA

## Abstract

Non-alcoholic steatohepatitis (NASH) is a global health concern without treatment. The challenge in finding effective therapies is due to the lack of good mouse models and the complexity of the disease, characterized by gene–environment interactions. We tested the susceptibility of seven mouse strains to develop NASH. The severity of the clinical phenotypes observed varied widely across strains. PWK/PhJ mice were the most prone to develop hepatic inflammation and the only strain to progress to NASH with extensive fibrosis, while CAST/EiJ mice were completely resistant. Levels of mitochondrial transcripts and proteins as well as mitochondrial function were robustly reduced specifically in the liver of PWK/PhJ mice, suggesting a central role of mitochondrial dysfunction in NASH progression. Importantly, the NASH gene expression profile of PWK/PhJ mice had the highest overlap with the human NASH signature. Our study exposes the limitations of using a single mouse genetic background in metabolic studies and describes a novel NASH mouse model with features of the human NASH.

## Teaser

Mice from different genetic backgrounds have widely diverse responses to the same metabolic challenges. Only PWK/PhJ mice show significant mitochondrial dysfunction and progression to fibrotic NASH that resembles human NASH. The PWK/PhJ strain is a novel NASH mouse model.

## Graphical Abstract

**Figure:**
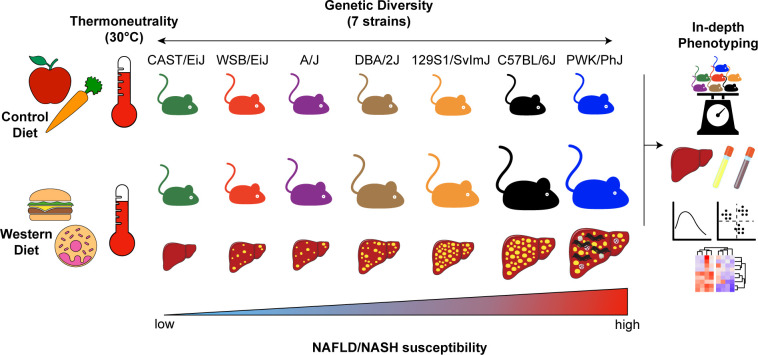

## Introduction

Non-alcoholic fatty liver disease (NAFLD) is the most prevalent chronic liver disease and is a global health burden, affecting about one in four people in western societies (Younossi et al. 2018; Younossi et al. 2016; Younossi and Henry 2022). NAFLD includes a wide range of liver disease stages that can vary from simple steatosis, which is usually benign and asymptomatic, to the more severe non-alcoholic steatohepatitis (NASH). NASH is a progressive liver disease characterized by steatosis, lobular inflammation, and cellular injury with or without fibrosis. A subset of patients with NASH may progress to extensive liver fibrosis, cirrhosis, hepatic failure, and hepatocellular carcinoma (Diehl and Day 2017; Dulai et al. 2017; Huang, El-Serag, and Loomba 2021; Sheka et al. 2020; Singh et al. 2015). Importantly, NASH is now one of the primary indications for liver transplantation (Wong et al. 2015). The disease constitutes a huge burden on society and continues to grow with the obesity and type II diabetes pandemics, two health conditions strongly associated with NAFLD (Chakravarthy and Neuschwander-Tetri 2020; Diehl and Day 2017). Given the robust association with metabolic diseases, NAFLD is regarded as the liver signature of the metabolic syndrome (Gastaldelli 2010). Consumption of excessive calories and sugar-rich diets have been linked to the development of the metabolic syndrome and its associated comorbidities, including NAFLD (Abdelmalek et al. 2010; Abid et al. 2009). However, human association studies have shown that the combined influence of environmental, genetic, and epigenetic factors is important to determine the onset and progression of NAFLD (Bayoumi et al. 2020; Chalasani et al. 2010; Du et al., 2021; Eslam and George 2016; Lee et al. 2017; Romeo et al. 2008; Speliotes et al. 2011). Not all patients with NAFLD will progress to NASH, and identifying patients at higher risk of disease progression is important to improve the management of patients.

Despite ongoing efforts to find effective therapies for NASH, the therapeutic options remain limited and there is still no Food and Drug Administration–approved drug for this disease (Filipovic et al. 2021; Shi and Fan 2021). Drug development for NASH has been hindered by the limited understanding of the disease and the lack of good animal models. NASH was once thought to be a “two hit” process, with excess lipid accumulation in the hepatocytes being the first hit and inflammation and fibrogenesis being the second hit (Day and James 1998). This view has more recently been challenged, and NASH is now recognized to be a complex multiple-hit process with several different mechanisms, tissues, and pathways playing a role in the progression of the disease (Buzzetti et al., 2016; Loomba et al.,2021). However, the molecular mechanisms underlying NAFLD progression are still incompletely understood.

Several animal models have been developed for NAFLD. They can be distinguished into dietary models, genetic models, toxicity models, or a combination of these. Each model has advantages and disadvantages, but none of them fully reproduces NASH in humans (Farrell et al. 2019; Hunter et al. 2020; Im et al. 2021; Santhekadur, Kumar, and Sanyal 2018). The struggle in finding appropriate animal models lies in the complexity of NAFLD pathophysiology. Dietary models aim to mimic the human metabolic syndrome and often develop hepatic steatosis and hyperglycemia, but they do not show progression to fibrosis, or they develop significant fibrosis only at a very late stage (Eng and Estall 2021). Fibrosis is a key histological feature of human NASH, a prognostic indicator in patients and an important endpoint in clinical trials of NASH (Denk et al.,2019). Toxicity models are rapid and develop steatosis and fibrosis, but also feature body weight loss and manifest no other aspect of the metabolic syndrome (Farrell et al. 2019). Similarly, genetic models of NAFLD/NASH, with mutation/ablation of a single gene, fail to phenocopy the complexity of the human disease that is driven by the interplay between environmental factors, epigenetic modifications, and multiple single-nucleotide polymorphisms (Eslam and George 2016).

Here we explored NASH susceptibility in mice from seven different genetic backgrounds, i.e., C57BL/6J, DBA/2J, A/J, 129S1/SvlmJ, WSB/EiJ, CAST/EiJ, and PWK/PhJ, that were fed a western-style diet (WD) and housed at thermoneutrality (TN). We found wide differences across mouse strains in nearly all phenotypes tested, with the PWK/PhJ mice being the most sensitive strain to NAFLD/NASH and the only strain to show progression to fibrotic NASH. The specific pathways downregulated in the liver of PWK/PhJ mice were related to mitochondria; the PWK/PhJ strain was furthermore the only strain with severely compromised mitochondrial function. Comparison of the mouse expression data with two publicly available human NASH datasets revealed that PWK/PhJ mice were also the closest to humans at the gene expression level. The PWK/PhJ strain hence constitutes a novel NASH mouse model that rapidly progresses to liver fibrosis, manifests features of the metabolic syndrome, and thus recapitulates several aspects of the human NASH.

## Results

### The mouse genetic background is a major determinant of the physiological responses to metabolic challenges

**Figure 1.**
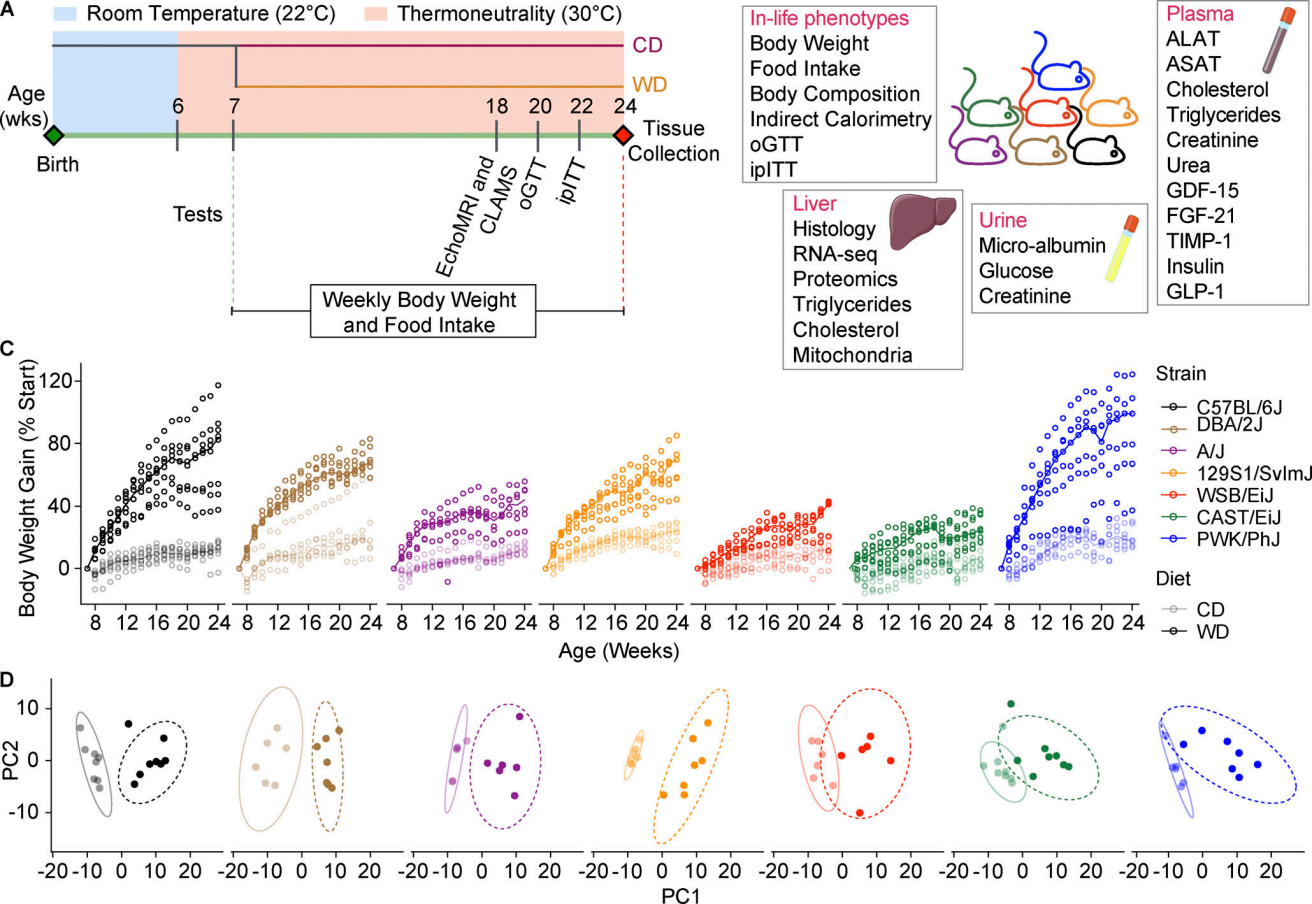
**The mouse genetic background is a major determinant of the physiological responses to metabolic challenges. (A)** Schematic of the experimental pipeline. Seven mouse strains (C57BL/6J, DBA/2J, A/J, 129S1/SvlmJ, WSB/EiJ, CAST/EiJ, PWK/PhJ) were housed at TN from 6 wk of age and fed a WD or CD from 7 wk of age. The in vivo phenotypes collected are indicated. **(B)** Overview of the in vivo, molecular, and biochemical phenotypes collected. **(C)** Body weight gain curves (expressed as percentage from the starting body weight) for each strain. The line represents the median. **(D)** PCA of all the in vivo and biochemical phenotypes collected for each strain separately. For all panels: C57BL/6J-CD *n* = 8, C57BL/6J-WD *n* = 8, DBA/2J-CD *n* = 6, DBA/2J-WD *n* = 7, A/J-CD *n* = 6, A/J-WD *n* = 6, 129S1/SvlmJ-CD *n* = 7, 129S1/SvlmJ-WD *n* = 7, WSB/EiJ-CD *n* = 6, WSB/EiJ-WD *n* = 6, CAST/EiJ-CD *n* = 8, CAST/EiJ-WD *n* = 8, PWK/PhJ-CD *n* = 6, PWK/PhJ-WD *n* = 7. Each group of mice was assayed in two independent cohorts.

**Figure S1.**
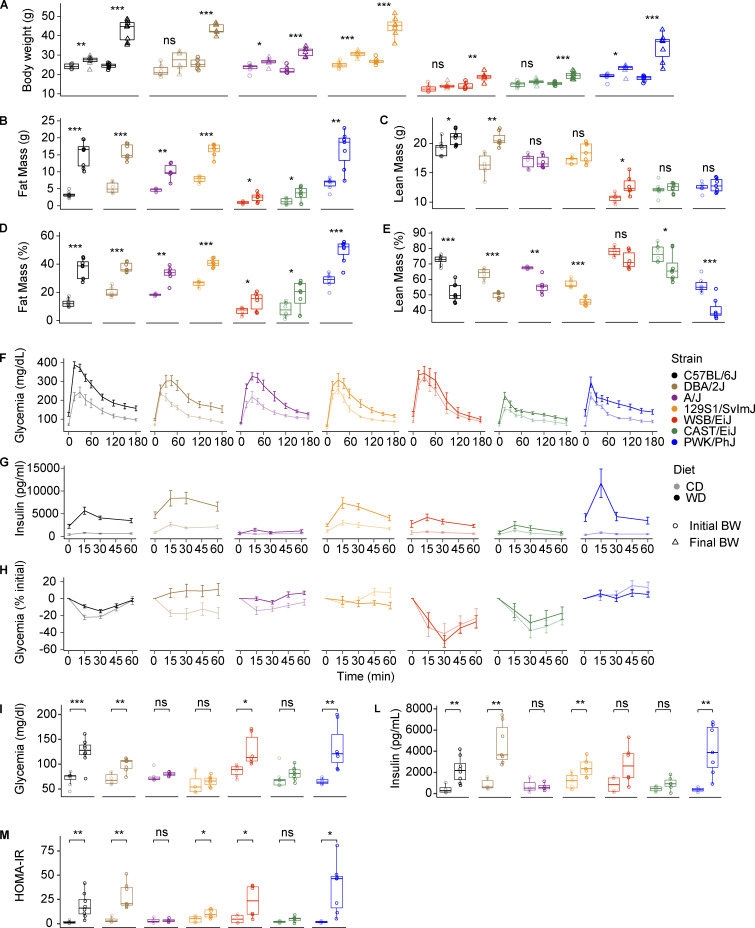
**The mouse genetic background is a major determinant of the physiological responses to metabolic challenges. (A)** Initial (circles) and final (triangles) body weight in grams on CD (light colors) and WD (dark colors). **(B and C)** Fat mass and lean mass on CD and WD for each strain expressed in grams. **(D and E)** Fat mass and lean mass on CD and WD for each strain expressed in percentage of the total body weight. **(F)** OGTT curves. **(G)** Glucose-stimulated insulin secretion measured during OGTT at the indicated timepoints. BW, body weight. **(H)** Insulin tolerance test. Glycemia levels are expressed as percentage of the initial glycemia. **(I)** Fasting glycemia measured after 12-h fasting. **(L)** Fasting insulin measured after 12-h fasting. **(M)** HOMA IR calculated from 12-h fasting insulin and glucose levels from I and L using the formula [fasting insulin (mU/liter) × fasting glucose (mmol/liter)]/22.5. In F–H, data are represented as mean ± SEM. In A–E and I–M, data are represented as box and whiskers. The lower and upper hinges correspond to the first and third quartiles (the 25th and 75th percentiles). The upper whisker extends from the hinge to the largest value no further than 1.5 × IQR from the hinge (where IQR is the interquartile range, or distance between the first and third quartiles). The lower whisker extends from the hinge to the smallest value at most 1.5 × IQR of the hinge. Data beyond the end of the whiskers are called “outlying” points and are plotted individually. Statistical analysis for A–E and I–M: pairwise *t* test adjusted for multiple testing. *, P < 0.05; **, P < 0.01; ***, P < 0.001. For all panels: C57BL/6J-CD *n* = 8, C57BL/6J-WD *n* = 8, DBA/2J-CD *n* = 6, DBA/2J-WD *n* = 7, A/J-CD *n* = 6, A/J-WD *n* = 6, 129S1/SvlmJ-CD *n* = 7, 129S1/SvlmJ-WD *n* = 7, WSB/EiJ-CD *n* = 6, WSB/EiJ-WD *n* = 6, CAST/EiJ-CD *n* = 8, CAST/EiJ-WD *n* = 8, PWK/PhJ-CD *n* = 6, PWK/PhJ-WD *n* = 7. Each group of mice was assayed in two independent cohorts.

To understand to what extent genetic differences may affect NASH development, we investigated NASH susceptibility in mice from seven different genetic backgrounds (C57BL/6J, DBA/2J, A/J, 129S1/SvlmJ, WSB/EiJ, CAST/EiJ, PWK/PhJ). Six of these strains are among the founders of the Collaborative Cross (CC; Welsh et al. 2012) and Diversity Outbred (Churchill et al. 2012) populations (C57BL/6J, A/J, 129S1/SvlmJ, WSB/EiJ, CAST/EiJ, and PWK/PhJ). DBA/2J was included in this panel because it is the founder, along with C57BL/6J, of the BXD recombinant inbred lines (Peirce et al. 2004; Taylor et al. 1999). With this selection, three distinct *Mus musculus* subspecies are represented: *Mus musculus musculus* (PWK/PhJ), *Mus musculus castaneous* (CAST/EiJ), and *Mus musculus domesticus* (the remaining strains; Phifer-Rixey and Nachman 2015). To mimic a metabolic syndrome–like phenotype and induce liver disease, mice were housed at TN starting at 6 wk of age and were fed a WD (WD-TN) or a matched control diet (CD-TN) starting from 7 wk of age for 17 wk (Fig. 1 A). Housing at TN, which for mice is 30°C (Ganeshan and Chawla 2017; Škop et al. 2020), was shown to be more effective than housing at room temperature (RT) to trigger liver damage (Giles et al. 2017). Body weight and food intake were monitored weekly. Body composition, energy expenditure, glucose, and insulin tolerance were measured starting from 11 wk after diet following the timeline shown in Fig. 1 A. After tissue collection, several biochemical, histological, and molecular measurements were performed in liver, plasma, and urine samples (Fig. 1 B; see Table S1 for the phenotypes explanation). The seven strains differed widely in the amount of weight gained after 17 wk of WD (Fig. 1 C and Fig. S1 A). The C57BL/6J and PWK/PhJ strains were the most sensitive to WD-induced body weight gain, while WSB/EiJ and CAST/EiJ mice showed only a modest body weight gain and were almost undistinguishable from CD-fed mice (Fig. 1 C and Fig. S1 A). The increase in body weight gain was mostly attributable to an increase in fat mass, while the increase in lean mass was modest or absent for most strains (Fig. S1, B and C). All strains had increased fat mass on WD compared to the CD control. However, the fat mass percentage at the end of the study differed widely across strains: PWK/PhJ had the highest fat mass percentage, while WSB/EiJ and CAST/EiJ had the lowest (Fig. S1, D–E).

**Figure 2.**
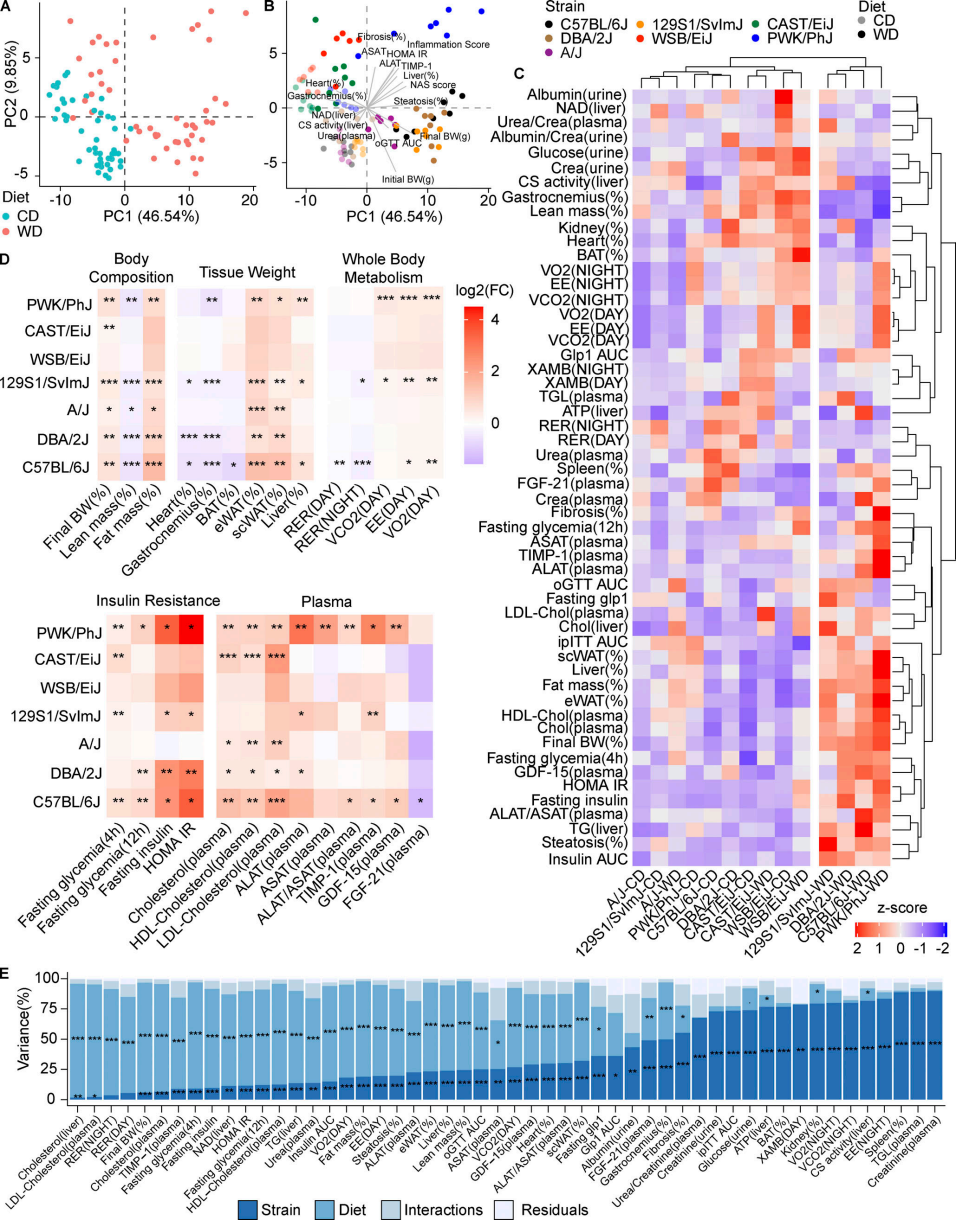
**The mouse genetic background is a major determinant of the physiological responses to metabolic challenges. (A and B)** PCA of all the in vivo and biochemical phenotypes collected. In A, dot colors identify the diet. In B, dot colors identify the strain. Lighter and darker colors indicate the diet (CD and WD, respectively). Vectors are shown for selected phenotypes. **(C)** Heatmap of z-scores of all phenotypes, experimental groups, and phenotypes are clustered based on the z-scores. **(D)** Heatmap of the log_2_-transformed fold changes for selected phenotypes for the comparison WD-CD for each strain. Significance levels are indicated. **(E)** Variance of phenotypes explained by strain, diet, their interaction, and the residuals of the linear model expressed as the mean sum of squares divided by the total mean sum of squares. Mean sum of squares is equal to the sum of squares divided by the degree of freedom of each parameter. Significance levels are indicated. Statistical analysis for D: Student’s *t* test with Benjamini–Hochberg adjusted P values. Statistical analysis for E: ANOVA with multiple testing-adjusted P value. *, P < 0.05; **, P < 0.01; ***, P < 0.001. For all panels: C57BL/6J-CD *n* = 8, C57BL/6J-WD *n* = 8, DBA/2J-CD *n* = 6, DBA/2J-WD *n* = 7, A/J-CD *n* = 6, A/J-WD *n* = 6, 129S1/SvlmJ-CD *n* = 7, 129S1/SvlmJ-WD *n* = 7, WSB/EiJ-CD *n* = 6, WSB/EiJ-WD *n* = 6, CAST/EiJ-CD *n* = 8, CAST/EiJ-WD *n* = 8, PWK/PhJ-CD *n* = 6, PWK/PhJ-WD *n* = 7. Each group of mice was assayed in two independent cohorts.

To obtain a global overview of the effects of WD-TN in each strain, we performed principal component analysis (PCA) on all the phenotypes collected (130) for each strain separately (Fig. 1 D). In all cases, the first principal component separates the mice based on the diet, indicating that all the strains responded to the diet to some extent (Fig. 1 D). PCA performed on all the phenotypes and strains together showed that mice were closer together on CD-TN, however when put on WD-TN they separated in three main groups (Fig. 2 A); PWK/PhJ, C57BL/6J, DBA/2J, and 129S1/SvlmJ mice on WD were separated from the rest of the mice along the first principal component (Fig. 2 B), explaining 46.54% of the variance. The second principal component, accounting for 9.85% of the variance, separated the strains based on the genetic background (wild-derived strains [PWK/PhJ, CAST/EiJ, and WSB/EiJ] vs. classical laboratory strains [C57BL/6J, DBA/2J, A/J, 129S1/SvlmJ]; Fig. 2 B). PWK/PhJ mice on WD-TN grouped separately from all other strains and conditions, and the phenotypes that drove this separation were related to liver damage and insulin resistance (e.g., homeostatic model assessment of insulin resistance [HOMA IR], alanine aminotransferase [ALAT], inflammation score, and liver weight). Hierarchical clustering of the strains based on the phenotype z-score confirmed this separation and identified two main clusters (Fig. 2 C). One cluster contained PWK/PhJ, C57BL/6J, DBA/2J, and 129S1/SvlmJ strains on WD, while the second contained all the strains on CD plus A/J, WSB/EiJ, and CAST/EiJ on WD. This indicated that PWK/PhJ, C57BL/6J, DBA/2J, and 129S1/SvlmJ were more severely affected by the diet, compared to A/J, WSB/EiJ, and CAST/EiJ, which were more similar to their respective CD controls. Among the sensitive strains, the PWK/PhJ mice were the most distant from all other strains (Fig. 2 C).

To further dissect how much selected phenotypes were affected by the diet in each strain, we calculated the log_2_ fold change for the WD vs. CD comparison (Fig. 2 D). While body weight–related parameters (e.g., final body weight gain, lean mass, and fat mass) were affected to a similar extent in PWK/PhJ, C57BL/6J, DBA/2J, and 129S1/SvlmJ strains, PWK/PhJ mice showed more severe insulin resistance and worsened liver damage–related phenotypes like liver weight and plasma ALAT and aspartate aminotransferase (ASAT) levels (Fig. 2 D and Fig. S1, F–M). In WSB/EiJ and CAST/EiJ mice, most of the measured phenotypes were not affected by diet, confirming that these strains were the most resistant to the WD-TN challenge (Fig. 2, C and D). Although both the genetic background and the diet significantly contributed to the phenotypic variance for most of the phenotypes (Fig. 2 E), for traits such as plasma triglycerides and creatinine levels, the variance was mostly explained by the genetic background. Conversely, other phenotypes such as liver and plasma cholesterol levels, respiratory exchange ratio, and final body weight gain were mostly determined by the diet (Fig. 2 E). In conclusion, our phenotypic analysis in seven different mouse strains identified a wide spectrum of responses to CD-TN and WD-TN with extremely sensitive and extremely resistant strains, as well as strains that were moderately affected by the metabolic challenges. The PWK/PhJ mice stand out of our analysis and the phenotypes that drove their separation from all other strains included liver damage and insulin resistance.

### PWK/PhJ mice are the most sensitive to liver damage and NASH progression to fibrosis

**Figure 3.**
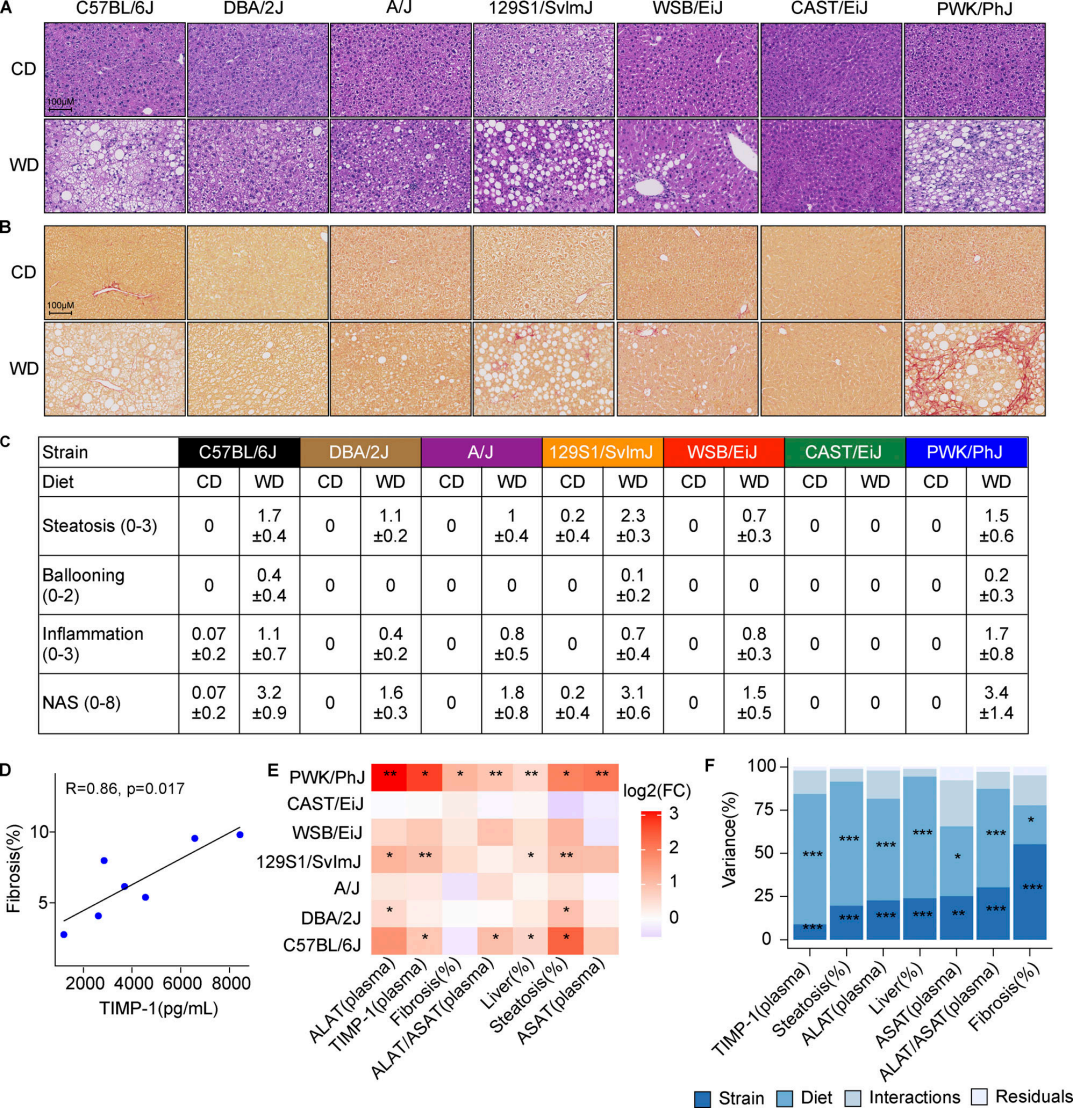
**PWK/PhJ mice are the most sensitive to liver damage and NASH progression to fibrosis. (A and B)** H&E (A) and Sirius red staining (B) of formalin-fixed liver sections (one representative image per strain). **(C)** Pathological scoring of liver H&E-stained sections. See Materials and methods for details regarding the scoring system and NAS calculation. **(D)** Correlation between liver fibrosis and plasma TIMP-1 levels in PWK/PhJ mice on WD-TN. Pearson R and P value are shown. **(E)** Heatmap of the log_2_-transformed fold changes for the comparison WD-CD of liver damage–related phenotypes for each strain. Significance levels are indicated. **(F)** Variance of liver damage phenotypes explained by strain, diet, their interaction, and the residuals of the linear model expressed as the mean sum of squares divided by the total mean sum of squares, as described for Fig. 2. Significance levels are indicated. Statistical analysis for E: Student’s *t* test with Benjamini–Hochberg adjusted P values. Statistical analysis for F: ANOVA with multiple testing-adjusted P value. For E and F: *, P < 0.05; **, P < 0.01; ***, P < 0.001. For all panels: C57BL/6J-CD *n* = 8, C57BL/6J-WD *n* = 8, DBA/2J-CD *n* = 6, DBA/2J-WD *n* = 7, A/J-CD *n* = 6, A/J-WD *n* = 6, 129S1/SvlmJ-CD *n* = 7, 129S1/SvlmJ-WD *n* = 7, WSB/EiJ-CD *n* = 6, WSB/EiJ-WD *n* = 6, CAST/EiJ-CD *n* = 8, CAST/EiJ-WD *n* = 8, PWK/PhJ-CD *n* = 6, PWK/PhJ-WD *n* = 7. Each group of mice was assayed in two independent cohorts.

**Figure S2.**
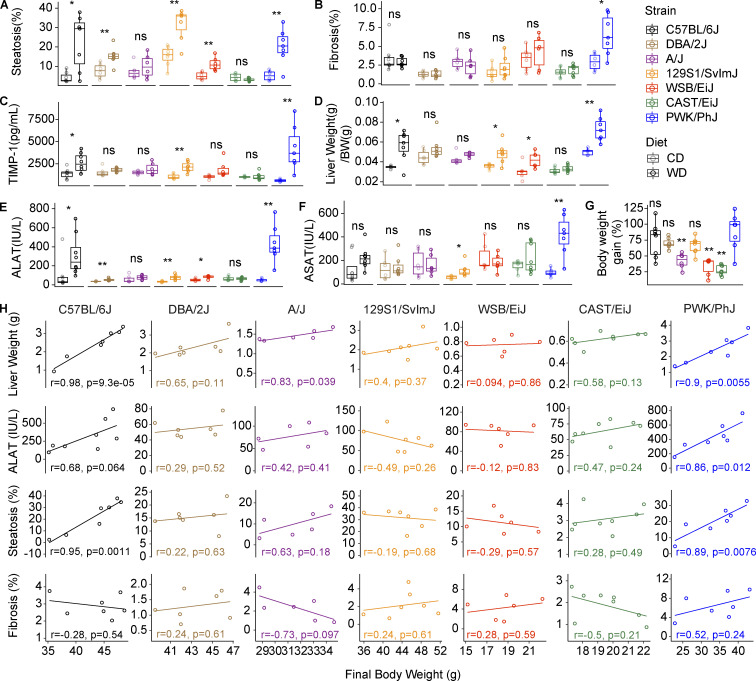
**PWK/PhJ mice are the most sensitive to liver damage and NASH progression to fibrosis. (A)** Steatosis percentage measured from H&E-stained liver sections. **(B)** Fibrosis percentage, measured as percentage of collagen content from Sirius red–stained liver sections. **(C)** Plasma TIMP-1 levels. **(D)** Liver weight normalized to body weight at sacrifice. **(E)** Plasma ALAT levels. **(F)** Plasma ASAT levels. **(G)** Final body weight gain on WD. Significance level is shown for the comparison of each strain with the PWK/PhJ strain. **(H)** Pairwise correlations of body weight with key liver damage phenotypes for each strain on WD-TN. Pearson *r* and P values are shown. In A–G, data are represented as box and whiskers. The lower and upper hinges correspond to the first and third quartiles (the 25th and 75th percentiles). The upper whisker extends from the hinge to the largest value no further than 1.5 × IQR from the hinge (where IQR is the interquartile range, or distance between the first and third quartiles). The lower whisker extends from the hinge to the smallest value at most 1.5 × IQR of the hinge. Data beyond the end of the whiskers are called “outlying” points and are plotted individually. Statistical analysis for A–G: Pairwise *t* test adjusted for multiple testing. *, P < 0.05; **, P < 0.01; ***, P < 0.001. For all panels: C57BL/6J-CD *n* = 8, C57BL/6J-WD *n* = 8, DBA/2J-CD *n* = 6, DBA/2J-WD *n* = 7, A/J-CD *n* = 6, A/J-WD *n* = 6, 129S1/SvlmJ-CD *n* = 7, 129S1/SvlmJ-WD *n* = 7, WSB/EiJ-CD *n* = 6, WSB/EiJ-WD *n* = 6, CAST/EiJ-CD *n* = 8, CAST/EiJ-WD *n* = 8, PWK/PhJ-CD *n* = 6, PWK/PhJ-WD *n* = 7. Each group of mice was assayed in two independent cohorts.

**Figure S3.**
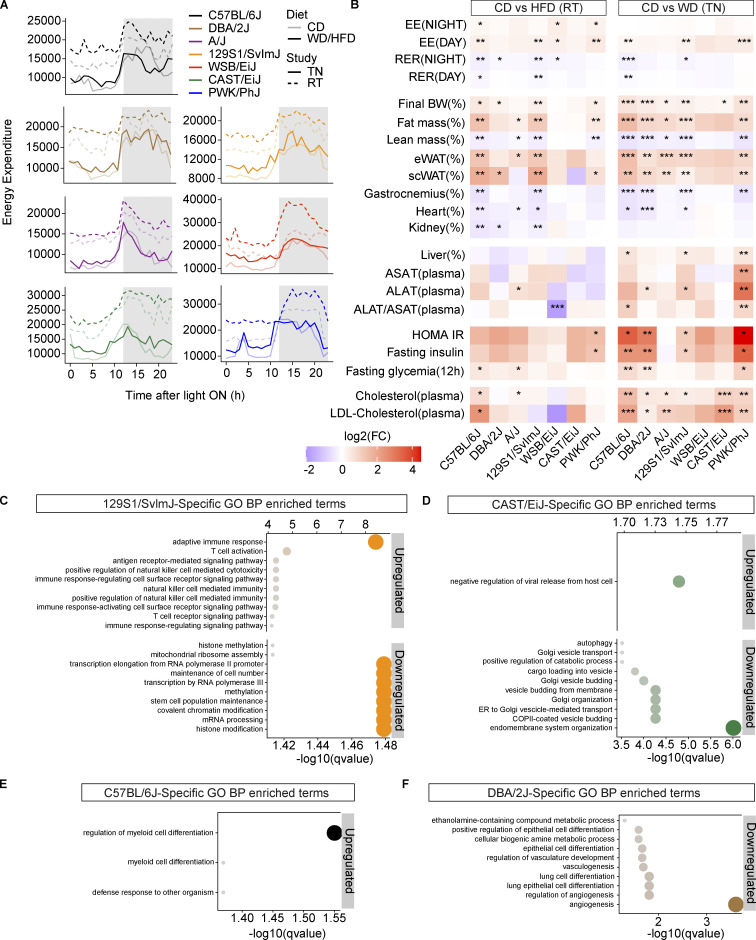
**Comparison of the effect of HFD-RT and WD-TN on different phenotypes and strain-specific ORA. (A)** Energy expenditure (hour averages) at TN (continuous lines) or RT housing (dotted lines). Lines indicate the median. Lighter and darker colors indicate the diet (CD or WD/HFD, respectively). **(B)** Heatmap of the log_2_-transformed fold changes of selected phenotypes for the comparison CD-HFD for each strain at RT (left) or TN (right). Significance levels are indicated. EE, energy expenditure; BW, body weight; RER, respiratory exchange ratio; eWAT, epidydimal white adipose tissue; scWAT, subcutaneous white adipose tissue. **(C–F)** Top 10 enriched GO-BP terms among 129S1/SvlmJ-specific (C), CAST/EiJ-specific (D), C57BL/6J-specific (E), and DBA/2J-specific (F) upregulated or downregulated genes on WD. Dot size indicates significance (−log10[qvalue]). For A and B: C57BL/6J-CD-TN *n* = 8, C57BL/6J-WD-TN *n* = 8, DBA/2J-CD-TN *n* = 6, DBA/2J-WD-TN *n* = 7, A/J-CD-TN *n* = 6, A/J-WD-TN *n* = 6, 129S1/SvlmJ-CD-TN *n* = 7, 129S1/SvlmJ-WD-TN *n* = 7, WSB/EiJ-CD-TN *n* = 6, WSB/EiJ-WD-TN *n* = 6, CAST/EiJ-CD-TN *n* = 8, CAST/EiJ-WD-TN *n* = 8, PWK/PhJ-CD-TN n = 6, PWK/PhJ-WD-TN *n* = 7, C57BL/6J-CD-RT *n* = 5, C57BL/6J-HFD-RT *n* = 5, DBA/2J-CD-RT *n* = 5, DBA/2J-HFD-RT *n* = 5, A/J-CD-RT *n* = 5, A/J-HFD-RT *n* = 5, 129S1/SvlmJ-CD-RT *n* = 5, 129S1/SvlmJ-HFD-RT *n* = 4, WSB/EiJ-CD-RT *n* = 5, WSB/EiJ-HFD-RT *n* = 5, CAST/EiJ-CD-RT *n* = 3, CAST/EiJ-HFD-RT *n* = 3, PWK/PhJ-CD-RT *n* = 5, PWK/PhJ-HFD-RT *n* = 5. Each group of mice was assayed in two independent cohorts. Statistical analysis for B: Student’s *t* test with Benjamini–Hochberg adjusted P values. *, P < 0.05; **, P < 0.01; ***, P < 0.001.

To better understand how genetic variability may influence the severity of the liver alterations, we performed H&E and Sirius red staining in the seven strains to measure fat accumulation and fibrosis development respectively (Fig. 3, A and B). Almost all strains, with the exception the CAST/EiJ, showed increased fat deposition on WD-TN (Fig. 3 A and Fig. S3 A). C57BL/6J, 129S1/SvlmJ, and PWK/PhJ mice, developed the most severe liver steatosis (Fig. 3 A and Fig. S2 A). However, PWK/PhJ mice were the only ones to develop significant fibrosis on WD-TN, as highlighted by increased collagen deposition, indicating a more advanced liver disease (Fig. 3 B and Fig. S2 B). Pathological scoring of liver H&E sections revealed that while all strains developed histological alterations to some degree, CAST/EiJ mice were completely resistant, and their livers were histologically undistinguishable from the CD-TN counterparts (Fig. 3 C). While 129S1/SvlmJ had the highest steatosis score, PWK/PhJ mice had the highest inflammation and NAFLD activity scores (NAS; Fig. 3 C).

PWK/PhJ was also the strain with the highest plasma TIMP-1 levels (Fig. S2 C). As seen in patients with chronic liver disease, TIMP-1 levels in PWK/PhJ mice were strongly correlated with fibrosis (Fig. 3 D; Murawaki et al. 1997; Rosenberg et al. 2004). Overall, PWK/PhJ mice had the most severe liver phenotype; all liver damage–related phenotypes were significantly affected in this strain, while CAST/EiJ were the most resistant (Fig. 3 E and Fig. S2, A–F). Since PWK/PhJ mice were among the strains that gained the most weight on WD-TN, we tested whether the increased liver damage was simply because PWK/PhJ mice were heavier compared the others. First, we found no significant differences in the final body weight gained between PWK/PhJ mice and C57BL/6J, 129S1/SvlmJ, or DBA/2J (Fig. S2 G). Second, while liver weight, plasma ALAT levels, and steatosis correlated with body weight in PWK/PhJ and C57BL/6J, fibrosis levels did not correlate with body weight (Fig. S2 H). Indeed, while for most liver damage phenotypes measured, the diet explained a higher proportion of the phenotypic variance, the genetic background was predominant in determining the variance in fibrosis (Fig. 3 F). This suggests that, while initial fat accumulation in the liver is primarily a consequence of environmental challenges, the likelihood to progress to fibrotic stages is predominantly determined by genetics. Of note, 129S1/SvlmJ mice developed the highest level of steatosis; however, steatosis was not correlated with body weight in this strain, indicating an important genetic contribution to liver fat accumulation for this strain (Fig. S2 H). Indeed, 129S1/SvlmJ mice had also the highest levels of steatosis on CD-TN compared to all other strains (Fig. S2 A). In summary, our analyses of the phenotypes induced by WD-TN identified a full range of responses. Based on these responses, the seven strains can be classified in four different groups: strains that are mostly resistant (CAST/EiJ, WSB/EiJ), strains with medium/low systemic response and a low degree of liver alterations (A/J, DBA/2J), strains with high systemic response and medium level of liver alterations (C57BL/6J, 129S1/SvlmJ), and strains with high systemic response and severe liver alterations with fibrosis (PWK/PhJ).

### TN exacerbates liver disease and insulin resistance without increasing body weight gain

**Figure 4.**
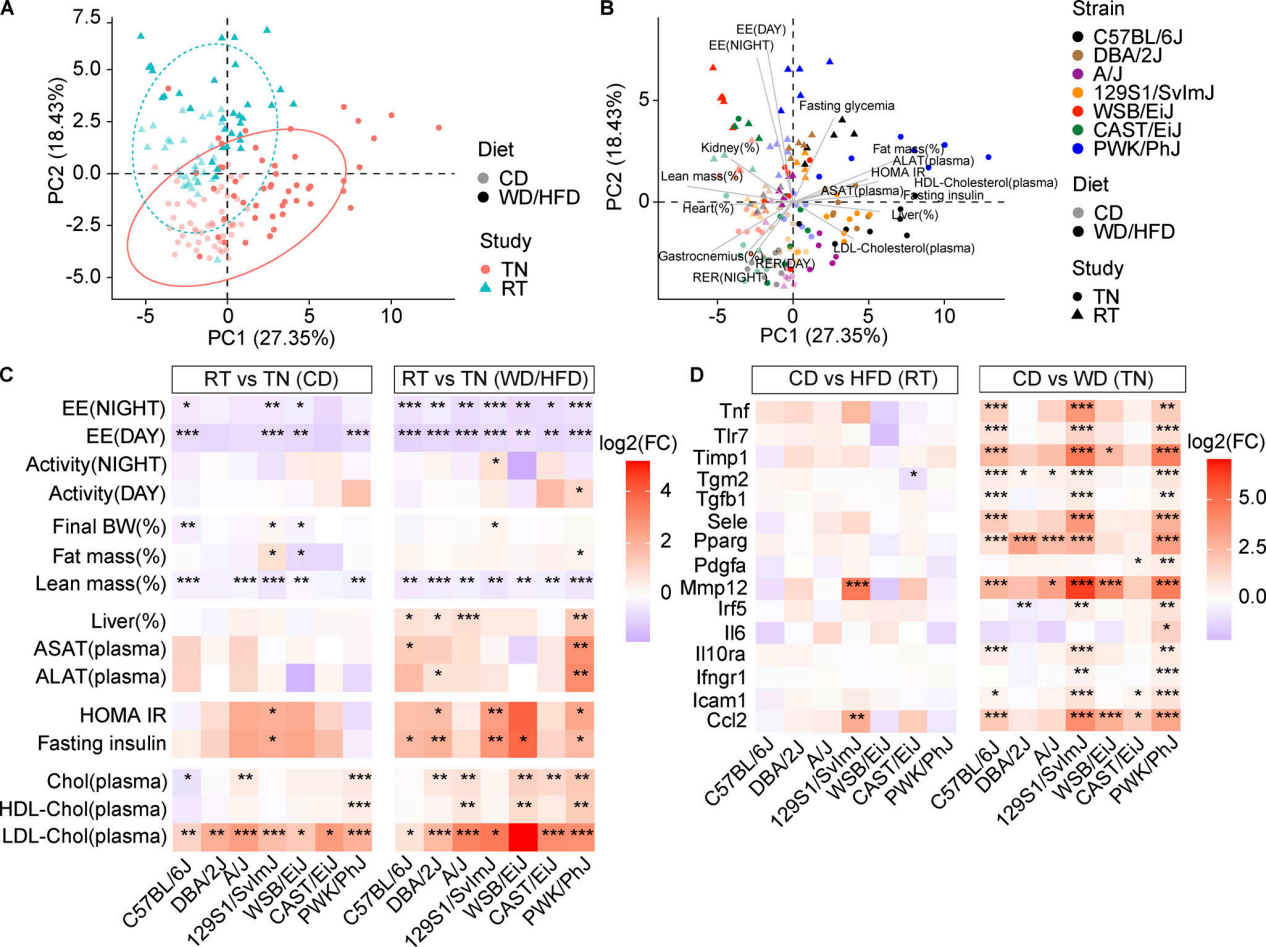
**TN exacerbates liver disease and insulin resistance without increasing body weight gain. (A)** PCA of common phenotypes collected for the TN and RT studies. Lighter and darker colors indicate the diet (CD or WD/HFD, respectively). Dot shape and color identifies the study. **(B)** PCA of common phenotypes collected for the TN and RT studies (same as in A) colored by strain. Lighter and darker colors indicate the diet (CD or WD/HFD, respectively). Dot shape identifies the study. Vectors are shown for selected phenotypes. **(C)** Heatmap of the log_2_-transformed fold changes of selected phenotypes for the comparison TN-RT for each strain on CD (left) or WD/HFD (right). EE, energy expenditure; BW, body weight. **(D)** Heatmap of the log_2_-transformed expression fold changes of inflammatory genes for the comparison CD-HFD/WD for each strain at RT (left) or TN (right). Significance levels are indicated. Statistical analysis for C and D: Student’s *t* test with Benjamini–Hochberg adjusted P values. *, P < 0.05; **, P < 0.01; ***, P < 0.001. For all panels: C57BL/6J-CD-TN *n* = 8, C57BL/6J-WD-TN *n* = 8, DBA/2J-CD-TN *n* = 6, DBA/2J-WD-TN *n* = 7, A/J-CD-TN *n* = 6, A/J-WD-TN *n* = 6, 129S1/SvlmJ-CD-TN *n* = 7, 129S1/SvlmJ-WD-TN *n* = 7, WSB/EiJ-CD-TN *n* = 6, WSB/EiJ-WD-TN *n* = 6, CAST/EiJ-CD-TN *n* = 8, CAST/EiJ-WD-TN *n* = 8, PWK/PhJ-CD-TN *n* = 6, PWK/PhJ-WD-TN *n* = 7, C57BL/6J-CD-RT *n* = 5, C57BL/6J-HFD-RT *n* = 5, DBA/2J-CD-RT *n* = 5, DBA/2J-HFD-RT *n* = 5, A/J-CD-RT *n* = 5, A/J-HFD-RT *n* = 5, 129S1/SvlmJ-CD-RT *n* = 5, 129S1/SvlmJ-HFD-RT *n* = 4, WSB/EiJ-CD-RT *n* = 5, WSB/EiJ-HFD-RT *n* = 5, CAST/EiJ-CD-RT *n* = 3, CAST/EiJ-HFD-RT *n* = 3, PWK/PhJ-CD-RT *n* = 5, PWK/PhJ-HFD-RT *n* = 5. Each group of mice was assayed in two independent cohorts.

To assess how our experimental setup (WD-TN) differs from more commonly used experimental setups (high-fat diet [HFD] at RT [HFD-RT]), we compared the phenotypes collected in our study (referred to as “TN study”) with those collected in a companion study from our group (Bachmann et al. 2022; referred to as “RT study”). PCA of the common phenotypes showed a clear separation of the two studies (Fig. 4 A) both on CD and WD/HFD. Energy expenditure and plasma cholesterol levels were among the phenotypes that contributed the most to this separation (Fig. 4 B). Indeed, all strains had higher low-density lipoprotein (LDL)–cholesterol when housed at TN both on CD and WD (Fig. 4 C). All strains had also lower energy expenditure when housed at TN and fed a WD both during the day and at night (Fig. 4 C and Fig. S3 A) when compared to RT housing, despite no differences being observed in total activity (Fig. 4 C). This suggested that lower energy expenditure in mice housed at TN was likely due to decreased thermogenesis rather than decreased locomotor activity.

The final body weight gain and final fat mass on WD/HFD were mostly unaffected by the study design (Fig. 4 C). Importantly, all strains had a lower lean mass when at WD-TN (Fig. 4 C). This decrease was also seen in most strains when comparing the CD groups at TN vs. RT (Fig. 4 C). Besides changes affecting all strains similarly, some strains showed worsening of NAFLD/NASH-related phenotypes like insulin resistance, liver weight, and liver enzymes levels in the TN study compared to the RT study (Fig. 4 C). Specifically, PWK/PhJ was the strain on which WD-TN has the strongest effect on liver weight and liver enzyme levels compared to HFD-RT, despite no differences being observed in the final body weight gain in the two studies (Fig. 4 C). This indicates that the experimental setup plays an important role in the severity of the liver phenotypes observed in this strain and confirms that the body weight gain is not driving the more extreme metabolic consequences observed in this strain on WD-TN.

A comparison within studies (Fig. S3 B) showed that WD-TN induced more robust changes and worsened most phenotypes in the sensitive strains (C57BL/6J, 129S1/SvlmJ, DBA/2J, and PWK/PhJ). Particularly worsened were insulin resistance, liver weight, liver enzymes levels, and plasma cholesterol levels, confirming our between-studies comparison (Fig. S3 B).

TN has been shown to accelerate inflammation in metabolic tissues like white adipose tissue (Tian et al. 2016). We hence compared the change of expression in inflammatory genes induced by HFD-RT and WD-TN (Fig. 4 D). Inflammatory genes were strongly induced by WD-TN, while they were mostly unaffected by HFD-RT. A link between metabolic inflammation and insulin resistance has been hypothesized (Wu and Ballantyne, 2022), and we indeed observed that insulin resistance was exacerbated by WD-TN.

Overall, insulin resistance, liver damage, and inflammation were particularly aggravated by WD-TN in the sensitive strains compared to HFD-RT conditions, despite final body weight gain and fat mass were similar between the two studies.

### The liver transcriptional response to WD-TN is strain specific

**Figure 5.**
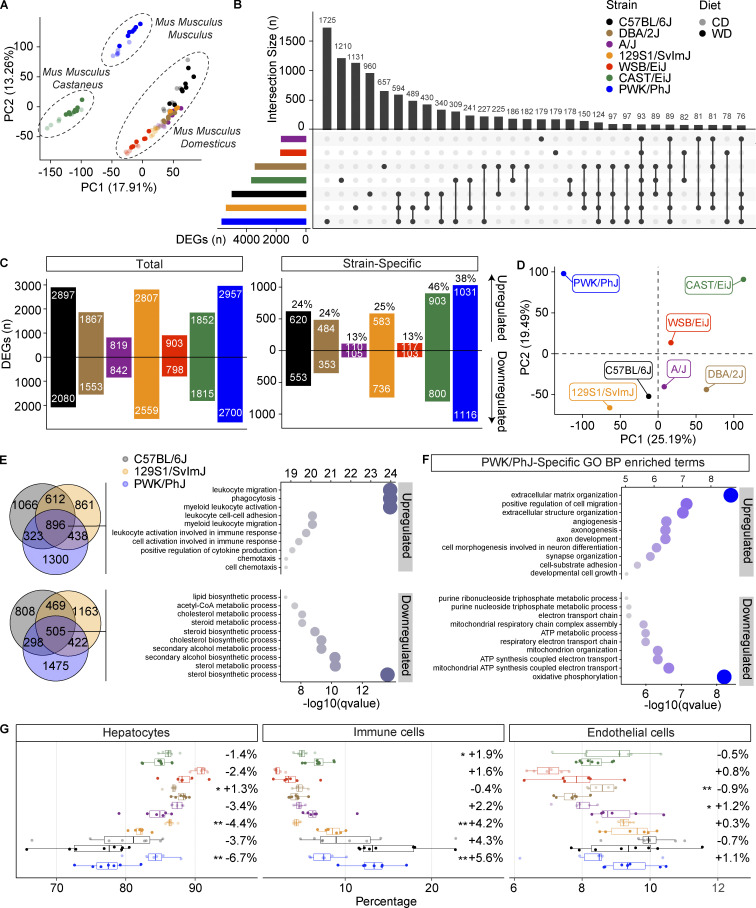
**The liver transcriptional response to WD-TN is strain specific. (A)** PCA of normalized liver gene expression data. Dot color indicates the strain. Lighter and darker colors indicate the diet (CD or WD, respectively). **(B)** Upset plot showing the total number of DEGs on WD in different mouse strains (colored bars in the bottom left corner) and number of genes that overlap between strains (black bars). The dots below each black bar indicate in which strain(s) the indicated number of genes are differentially expressed. **(C)** Left: Total number of genes significantly upregulated or downregulated on WD in the liver for each strain. Right: Number of genes significantly upregulated and downregulated only in one strain (strain-specific) in the liver on WD; percentages above each bar represent the proportion of strain-specific DEGs. **(D)** PCA on the liver gene expression log_2_ fold changes of WD vs. CD. **(E)** Left: Venn diagram showing the number of upregulated (upper panel) or downregulated (lower panel) genes on WD overlapping between the three strains that are most sensitive to the WD challenge (PWK/PhJ, C57BL/6J, 129S1/SvlmJ). Right: Top 10 enriched GO-BP among the upregulated (896) or downregulated (505) genes on WD common to the three sensitive strains. Dot size indicates significance (−log_10_[qvalue]). **(F)** Top 10 enriched GO-BP among PWK/PhJ-specific upregulated or downregulated genes on WD. Dot size indicates significance (−log10[qvalue]). (**G)** Single-cell deconvolution results indicating the estimated percentage of cell types in the mice liver. The percentages next to the boxplots indicate the change in average cell type proportion between WD and CD. In G, data are represented as box and whiskers. The lower and upper hinges correspond to the first and third quartiles (the 25th and 75th percentiles). The upper whisker extends from the hinge to the largest value no further than 1.5 × IQR from the hinge (where IQR is the interquartile range, or distance between the first and third quartiles). The lower whisker extends from the hinge to the smallest value at most 1.5 × IQR of the hinge. Data beyond the end of the whiskers are called "outlying" points and are plotted individually. Statistical analysis for G: Student’s *t* test with multiple testing correction. *, P < 0.05; **, P < 0.01; ***, P < 0.001. For all panels: C57BL/6J-CD *n* = 8, C57BL/6J-WD *n* = 8, DBA/2J-CD *n* = 6, DBA/2J-WD *n* = 7, A/J-CD *n* = 6, A/J-WD *n* = 6, 129S1/SvlmJ-CD *n* = 7, 129S1/SvlmJ-WD *n* = 7, WSB/EiJ-CD *n* = 6, WSB/EiJ-WD *n* = 6, CAST/EiJ-CD *n* = 8, CAST/EiJ-WD *n* = 8, PWK/PhJ-CD *n* = 6, PWK/PhJ-WD *n* = 7. Each group of mice was assayed in two independent cohorts.

To dissect the molecular mechanisms underlying different phenotypic responses to WD-TN, we performed liver RNA sequencing (RNA-seq) in the seven strains. PCA performed on gene expression levels separated the strains based on the subspecies (*M. musculus musculus* [PWK/PhJ], *M. musculus castaneous* [CAST/EiJ], and *M. musculus domesticus* [all other strains]; Fig. 5 A); this was expected, given the large genetic distance between them. Differential expression analysis revealed that the strains that had the highest steatosis levels on WD-TN (C57BL/6J, 129S1/SvlmJ, and PWK/PhJ; Fig. S2 A) also had the highest number of differentially expressed genes (DEGs; Fig. 5 B, colored bars, and Fig. 5 C, left panel) on WD-TN, in line with our phenotyping results. Among the DEGs, only 93 were common to all 7 strains, indicating that the transcriptional response was predominantly strain specific (Fig. 5 B, black bars). All the strains had roughly the same proportion of up- and downregulated genes (Fig. 5 C, left panel). PWK/PhJ and CAST/EiJ had the highest number of strain-specific up- and downregulated genes (Fig. 5 C, right), suggesting an effect of the subspecies in the response to the diet. Importantly, these two strains (PWK/PhJ and CAST/EiJ) were also the most sensitive and the most resistant to liver damage, respectively.

PCA performed on the diet-induced expression fold changes showed a separation of the strains based on the severity of the diet outcome on the liver along the first principal component, with sensitive strains (C57BL/6J, 129S1/SvlmJ, and PWK/PhJ) separating from all other strains. The second principal component separated the strains based on the genetic background with the wild-derived strains (PWK/PhJ, CAST/EiJ, and WSB/EiJ) separating from all other strains (Fig. 5 D). PWK/PhJ and CAST/EiJ strains were further apart from all other strains, reflecting the large genetic distance between mouse subspecies. To characterize the common molecular responses associated with susceptibility to WD-TN, we performed over-representation analysis (ORA) on overlapping up- (896) and downregulated (505) genes in the three most sensitive strains (C57BL/6J, 129S1/SvlmJ, and PWK/PhJ; Fig. 5 E). The 10 most significant (alpha = 0.05) upregulated gene ontologies for biological processes (GO-BP) exclusively included terms related to immune response activation, like leukocyte migration, adhesion and activation, and cytokine production (Fig. 5 E). On the other hand, the 10 most significantly downregulated gene ontologies included terms related to lipid and cholesterol metabolism (Fig. 5 E).

We then sought to characterize the strain-specific molecular responses associated with susceptibility and resistance to WD-TN. The PWK/PhJ strain had the most impacted transcriptome in terms of number of DEGs (Fig. 5, B and C) and was also the only strain to develop fibrosis. We thus we took an unbiased approach and looked at which pathways were enriched among the PWK/PhJ-specific DEGs. In line with our histology results, the most significant (alpha = 0.05) upregulated GO-BP terms were those related to fibrosis (extracellular matrix organization, cell migration, and extracellular structure organization; Fig. 5 F). Importantly, the 10 most significant downregulated GO-BP terms were all related to mitochondria and included oxidative phosphorylation (OXPHOS), ATP synthesis, and mitochondrial respiratory chain complex assembly (Fig. 5 F). This indicated that while inflammation and lipid metabolism are commonly altered in less severe liver disease stages, mitochondrial dysfunction underlies more severe liver disease, and fibrosis progression. We then performed ORA on the strain-specific DEGs for all the other strains (Fig. S3, C–F). The CAST/EiJ-specific downregulated gene ontologies included Golgi organization, Golgi vesicle budding, and ER to Golgi vesicle–mediated transport, while 129S1/SvlmJ-specific downregulated gene ontologies included histone and chromatin modification, methylation, transcription elongation, and mRNA processing. Given the small number of strain-specific DEGs, no significantly enriched gene ontologies were identified for WSB/EiJ and A/J.

We then inferred the impact of WD-TN on the liver cellular composition for each strain using single-cell deconvolution on the liver RNA-seq data (Fig. 5 G; see also Materials and methods). We observed an increase of immune cells proportion in most strains; however, the most sensitive strains showed the highest increase: PWK/PhJ (+5.6%), C57BL/6J (+4.3%), and 129S1/SvlmJ (+4.2%). Among the sensitive strains, PWK/PhJ had the highest increase in immune cells, confirming the histological scoring (Fig. 3 C). Accordingly, the largest decrease in hepatocytes proportion was observed for the same strains (Fig. 5 G).

### WD-TN induces a strong downregulation in the mitochondrial electron transport chain in PWK/PhJ mice

**Figure 6.**
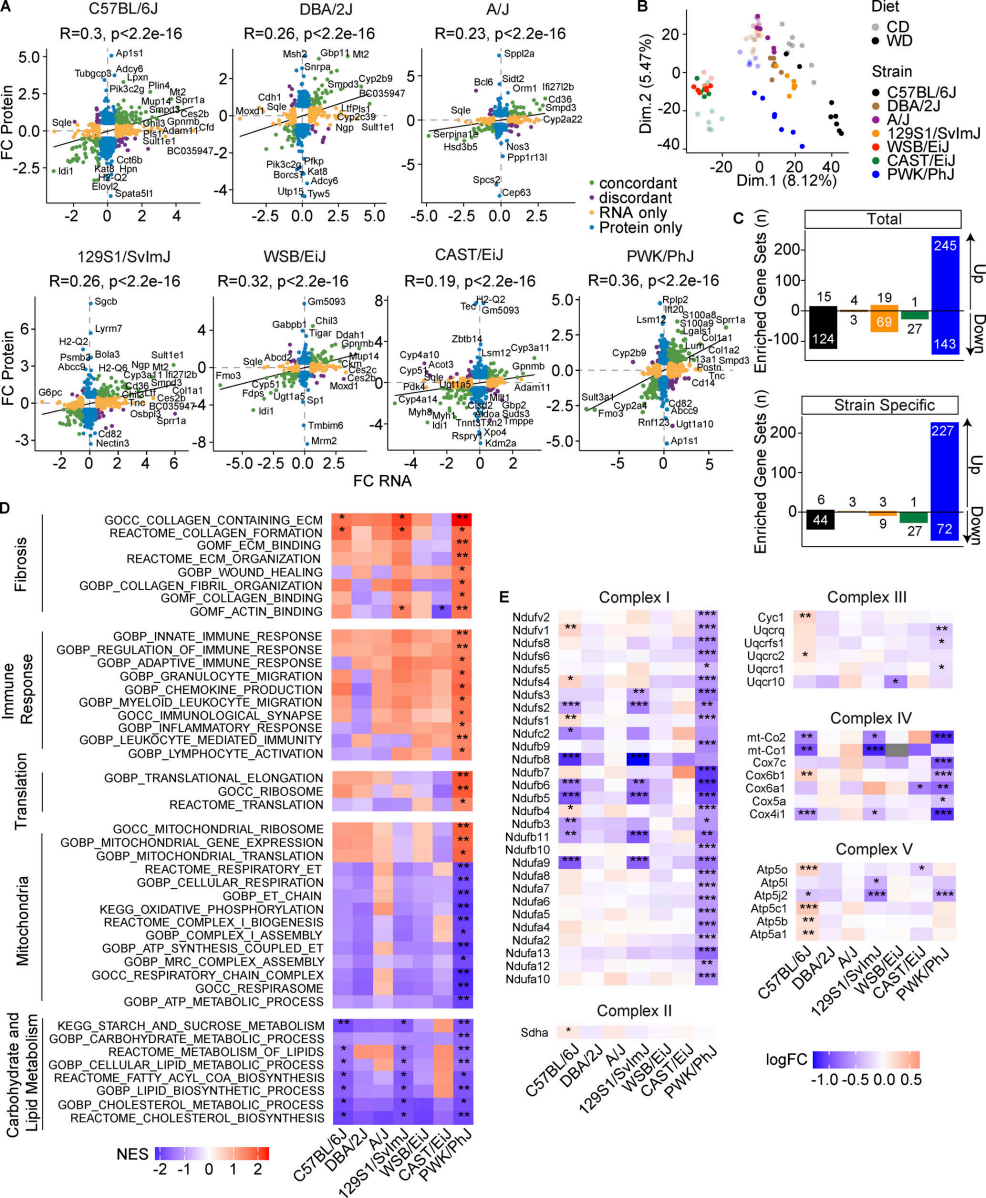
**WD-TN induces a strong downregulation in the mitochondrial electron transport chain in PWK/PhJ mice. (A)** Correlation between gene log_2_ fold changes (FC) of RNA and protein for the comparison CD vs. WD of all strains combined. Pearson *r* and P value are shown. **(B)** PCA on normalized protein levels. **(C)** Number of significantly enriched gene sets among the differentially expressed proteins per strain (only the strains with at least one significantly enriched gene set are shown). **(D)** Representative gene sets enriched in PWK/PhJ mice. **(E)** Log_2_ fold changes of electron transport chain complex subunits (only subunits that were differentially expressed in at least one strain are shown). For all panels: C57BL/6J-CD *n* = 8, C57BL/6J-WD *n* = 8, DBA/2J-CD *n* = 6, DBA/2J-WD *n* = 7, A/J-CD *n* = 6, A/J-WD *n* = 6, 129S1/SvlmJ-CD *n* = 5, 129S1/SvlmJ-WD *n* = 7, WSB/EiJ-CD *n* = 5, WSB/EiJ-WD *n* = 6, CAST/EiJ-CD *n* = 7, CAST/EiJ-WD *n* = 5, PWK/PhJ-CD *n* = 6, PWK/PhJ-WD *n* = 7. Each group of mice was assayed in two independent cohorts. Statistical analysis for D and E: Student’s *t* test with Benjamini–Hochberg adjusted P values. *, P < 0.05; **, P < 0.01; ***, P < 0.001. ECM, extracellular matrix; ET, electron transport; MRC, mitochondrial respiratory chain.

Our transcriptome analysis informed us on broad changes in the transcriptional program of the liver under WD-TN. Strains that were more sensitive to the environmental challenges had more profound transcriptional changes, while extremely sensitive and resistant strains had the highest number of strain-specific DEGs. While mRNA expression levels are often considered a surrogate of protein levels, it has been shown by multiple studies that mRNA expression changes only explain a small portion of the variability in protein levels (Vogel and Marcotte, 2012), in particular in response to environmental perturbations that lead to oxidative stress (Vogel et al., 2011). Therefore, to corroborate and complement our transcriptome results, we performed proteomics analysis in the liver of the seven strains on CD-TN and WD-TN. We first compared the log_2_ fold changes of the WD vs. CD comparison for mRNA and protein and found a significant positive correlation in all the strains (Fig. 6 A). PWK/PhJ mice had the highest correlation coefficient (0.36), while CAST/EiJ had the lowest (0.19). The overall low correlation coefficients were due to the fact that for several genes the effect of the WD-TN only impacted the mRNA (Fig. 6 A; RNA only) or the protein level (Fig. 6 A; protein only), but not both. To obtain a global overview of the effect of WD-TN on the proteome of each strain, we performed PCA on the normalized protein abundance. The mice grouped by strain and condition, confirming the good quality of the sample preparation and proteomics data analysis (Fig. 6 B). The diet had the strongest effect on the proteome of C57BL/6J and PWK/PhJ mice, as shown by the greater distance of the CD and WD groups for these strains. To investigate which pathways were most affected at the protein level, we performed gene set enrichment analysis (GSEA) on the differentially expressed proteins. PWK/PhJ mice had the highest number of total and strain-specific significantly enriched gene sets, while CAST/EiJ, DBA/2J, A/J, and WSB/EiJ had little or no enriched gene sets (Fig. 6 C; only strains with at least one enriched gene set are shown). Upregulated enriched gene sets in PWK/PhJ mice included fibrosis, immune response, and translation, while downregulated gene sets were related to carbohydrate, lipid and cholesterol metabolism and mitochondrial complex assembly, electron transport chain, and OXPHOS (Fig. 6 D). While carbohydrate and lipid metabolism were downregulated in the three most sensitive strains (C57BL/6J, 129S1/SvlmJ, and PWK/PhJ), mitochondria-related gene sets were specifically downregulated in PWK/PhJ mice, confirming our transcriptome results (Fig. 6 D). To dissect which element of the electron transport chain was most affected, we looked at the protein expression levels of different complex subunits. We found that complex I and complex IV were the most affected complexes (Fig. 6 E). More than 60% of complex I subunits (27/44), 36% of complex IV subunits (7/19), and 30% of complex III subunits (3/10) were downregulated in PWK/PhJ mice. No differences were found for complex II, and only one subunit of complex V was downregulated (Fig. 6 E). In conclusion, our proteome analysis revealed that WD had the highest impact on the proteome of PWK/PhJ mice and confirmed our transcriptome results: strong downregulation of mitochondria-related ontologies including complex biogenesis, mitochondrial electron transport, and OXPHOS were specifically downregulated in PWK/PhJ mice.

### PWK/PhJ mice have severe mitochondrial dysfunction on WD-TN

**Figure 7.**
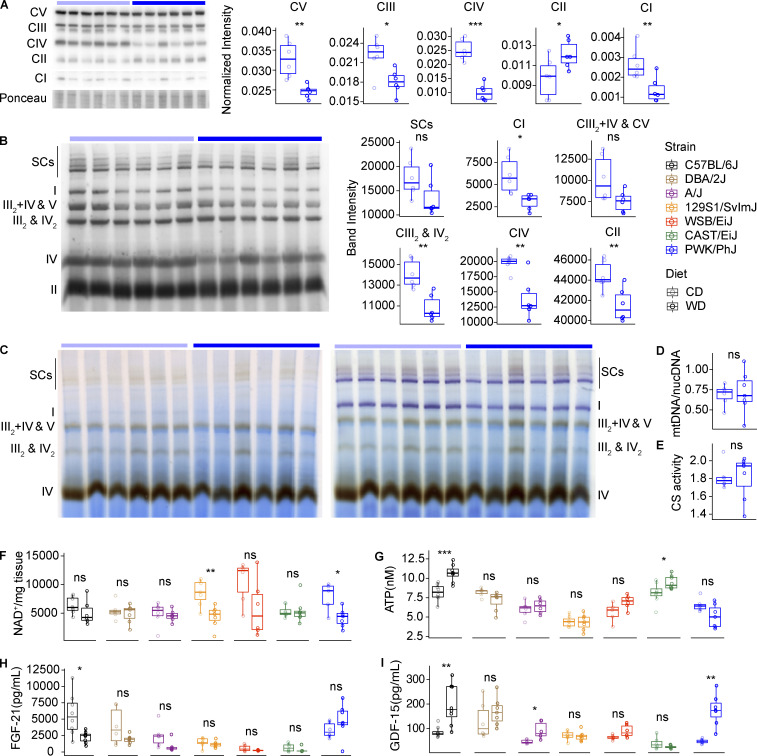
**PWK/PhJ mice have severe mitochondrial dysfunction on WD-TN. (A)** Western blot on isolated mitochondria from the liver with immunostaining of the five OXPHOS complexes (left) and band densitometry quantification normalized to ponceau staining (right panels) in PWK/PhJ mice. **(B)** Left: BN-PAGE on liver-isolated mitochondria from PWK/PhJ mice with immunostaining of the five OXPHOS complexes. Mitochondria respiratory complexes and supercomplexes corresponding to each band are indicated. Right: Band densitometry quantification of the indicated complexes and supercomplexes. **(C)** In-gel activity of complex IV (left, brown bands) and complex IV + complex I (right, brown bands and purple bands, respectively) in liver-isolated mitochondria from PWK/PhJ mice. Respiratory complexes and supercomplexes are indicated. **(D)** Liver mtDNA/nucDNA ratio. **(F)** Liver NAD^+^ quantification. **(E)** Liver citrate synthase activity quantification. **(G)** Liver ATP levels. **(H and I)** FGF-21 (H) and GDF-15 (I) plasma levels measured at the end of the study using the Luminex system (see Materials and methods). Data are represented as box and whiskers. The lower and upper hinges correspond to the first and third quartiles (the 25th and 75th percentiles). The upper whisker extends from the hinge to the largest value no further than 1.5 × IQR from the hinge (where IQR is the interquartile range, or distance between the first and third quartiles). The lower whisker extends from the hinge to the smallest value at most 1.5 × IQR of the hinge. Data beyond the end of the whiskers are called "outlying" points and are plotted individually. For panels A–C: PWK/PhJ-CD *n* = 6, PWK/PhJ-WD *n* = 6; for panels D–E: PWK/PhJ-CD *n* = 6, PWK/PhJ-WD *n* = 7; for panels F–I: C57BL/6J-CD *n* = 8, C57BL/6J-WD *n* = 8, DBA/2J-CD *n* = 6, DBA/2J-WD *n* = 7, A/J-CD *n* = 6, A/J-WD *n* = 6, 129S1/SvlmJ-CD *n* = 7, 129S1/SvlmJ-WD *n* = 7, WSB/EiJ-CD *n* = 6, WSB/EiJ-WD *n* = 6, CAST/EiJ-CD *n* = 8, CAST/EiJ-WD *n* = 8, PWK/PhJ-CD *n* = 6, PWK/PhJ-WD *n* = 7. Each group of mice was assayed in two independent cohorts. Statistical analysis for A–I: Student’s pairwise *t* test adjusted for multiple testing: *, P < 0.05; **, P < 0.01; ***, P < 0.001. Source data are available for this figure: SourceData F7.

**Figure S4.**
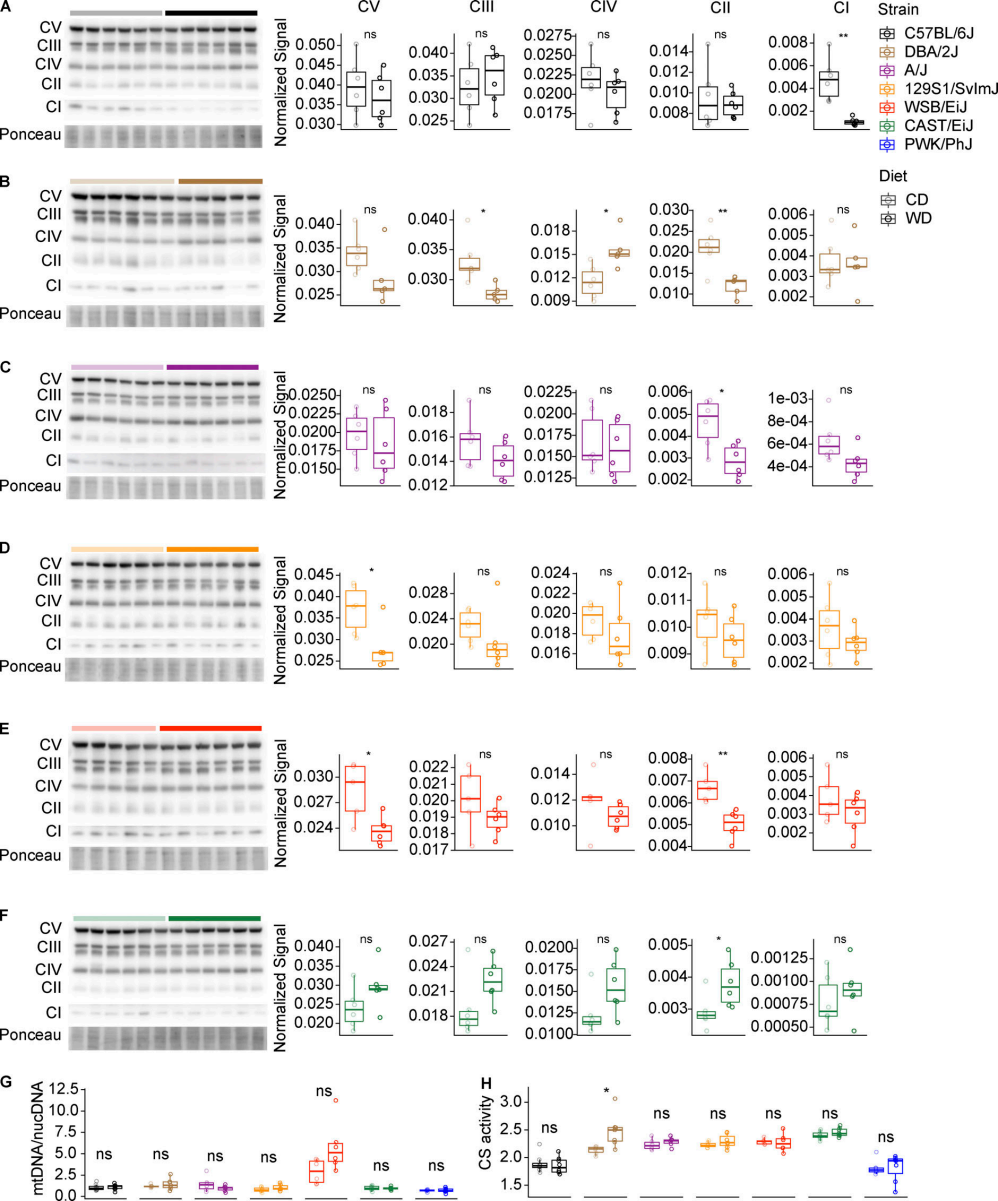
**PWK/PhJ mice have severe mitochondrial dysfunction on WD-TN. (A–F)** Western blot on liver-isolated mitochondria with immunostaining of the five OXPHOS complexes (left) and band densitometry quantification normalized to ponceau staining (right panels) in C57BL/6J (A), DBA/2J (B), A/J (C), 129S1/SvlmJ (D), WSB/EiJ (E), and CAST/EiJ (F) mice. **(G)** mtDNA to nucDNA ratio (average of three different mitochondrial genes [16S, ND1, and dloop] and HK2 nuclear gene) measured in the liver. **(H)** Citrate synthase activity measured in liver protein extracts. Data are represented as box and whiskers. The lower and upper hinges correspond to the first and third quartiles (the 25th and 75th percentiles). The upper whisker extends from the hinge to the largest value no further than 1.5 × IQR from the hinge (where IQR is the interquartile range, or distance between the first and third quartiles). The lower whisker extends from the hinge to the smallest value at most 1.5 × IQR of the hinge. Data beyond the end of the whiskers are called “outlying” points and are plotted individually. For A–F: C57BL/6J-CD *n* = 6, C57BL/6J-WD *n* = 6, DBA/2J-CD *n* = 6, DBA/2J-WD *n* = 5, A/J-CD *n* = 6, A/J-WD *n* = 6, 129S1/SvlmJ-CD *n* = 6, 129S1/SvlmJ-WD *n* = 6, WSB/EiJ-CD *n* = 5, WSB/EiJ-WD *n* = 6, CAST/EiJ-CD *n* = 6, CAST/EiJ-WD *n* = 6. For G–H: C57BL/6J-CD *n* = 8, C57BL/6J-WD *n* = 8, DBA/2J-CD *n* = 6, DBA/2J-WD *n* = 7, A/J-CD *n* = 6, A/J-WD *n* = 6, 129S1/SvlmJ-CD *n* = 7, 129S1/SvlmJ-WD *n* = 7, WSB/EiJ-CD *n* = 6, WSB/EiJ-WD *n* = 6, CAST/EiJ-CD *n* = 8, CAST/EiJ-WD *n* = 8, PWK/PhJ-CD *n* = 6, PWK/PhJ-WD *n* = 7. Statistical analysis for all panels: pairwise *t* test adjusted for multiple testing: *, P < 0.05; **, P < 0.01. Source data are available for this figure: SourceData FS4.

The strong downregulation of mitochondrial mRNA and protein expression in PWK/PhJ mice prompted us to characterize mitochondrial content, function, complex, and supercomplex assembly in the seven strains. First, we measured mitochondrial complexes by Western blot using a total OXPHOS antibody cocktail (ab110413; Abcam). This cocktail is optimized to quantify assembled mitochondrial complexes, since each antibody is against a subunit that is unstable when the complex is not assembled. We found that, in PWK/PhJ mice, most mitochondrial complexes were downregulated on WD-TN, in particular complex IV and complex I, which was consistent with the proteomics data (Fig. 7 A). This was specific of PWK/PhJ mice, as we did not observe a similar downregulation in any of the other six strains (Fig. S4, A–F). Complex II was the only complex to be upregulated in PWK/PhJ mice on WD-TN, likely because of a compensatory mechanism. Complex IV and complex I are main constituents of mitochondrial supercomplexes; we thus performed blue native polyacrylamide gel electrophoresis (BN-PAGE) on isolated mitochondria and measured the amount of supercomplexes and isolated complexes. Both isolated and superassembled complex I and IV were decreased in PWK/PhJ mice on WD-TN (Fig. 7 B). This decrease is thus likely due to the overall reduction in the amount of single complexes, rather than a reduction in the superassembly itself. To assess the enzymatic activity of mitochondrial complexes on WD-TN in PWK/PhJ, we performed in-gel activity for complex IV and I on isolated mitochondria from mouse livers and found that both complex I and IV had reduced activity on WD-TN in PWK7PhJ mice (Fig. 7 C).

While mitochondrial gene expression, protein expression, complex assembly, and function were strongly downregulated in PWK/PhJ mice, the mitochondria content was unchanged: PWK/PhJ mice on CD-TN and WD-TN had comparable mitochondrial DNA (mtDNA) to nuclear DNA (nucDNA) ratio and citrate synthase activity, two measurements used to estimate mitochondrial mass (Fig. 7, D and E). Similarly, we did not observe significant changes in mitochondria content upon WD-TN in the other six strains (Fig. S4, G and H). To further assess OXPHOS function, we measured NAD^+^ and ATP levels in the liver. PWK/PhJ was the only strain to show concomitant reduction of both NAD^+^ and ATP levels on WD (Fig. 7, F and G). Given the specific mitochondrial dysfunction observed in PWK/PhJ mice, we measured the plasma levels of two hepatokines, FGF-21 and GDF-15, that are also known as “mitokines” (i.e., they are released in the circulation upon mitochondrial stress). We found that only in PWK/PhJ mice, both FGF-21 and GDF-15 were concomitantly elevated by WD-TN, while FGF-21 was mostly decreased by WD-TN in the other strains (Fig. 7, H and I). Thus, our data suggest that PWK/PhJ mice may be more sensitive to overnutrition-induced mitochondrial damage. This may eventually lead to the accumulation of oxidative stress, cell death, inflammation, and fibrosis.

### The PWK/PhJ transcriptional response to WD-TN recapitulates changes seen in human NASH

**Figure 8.**
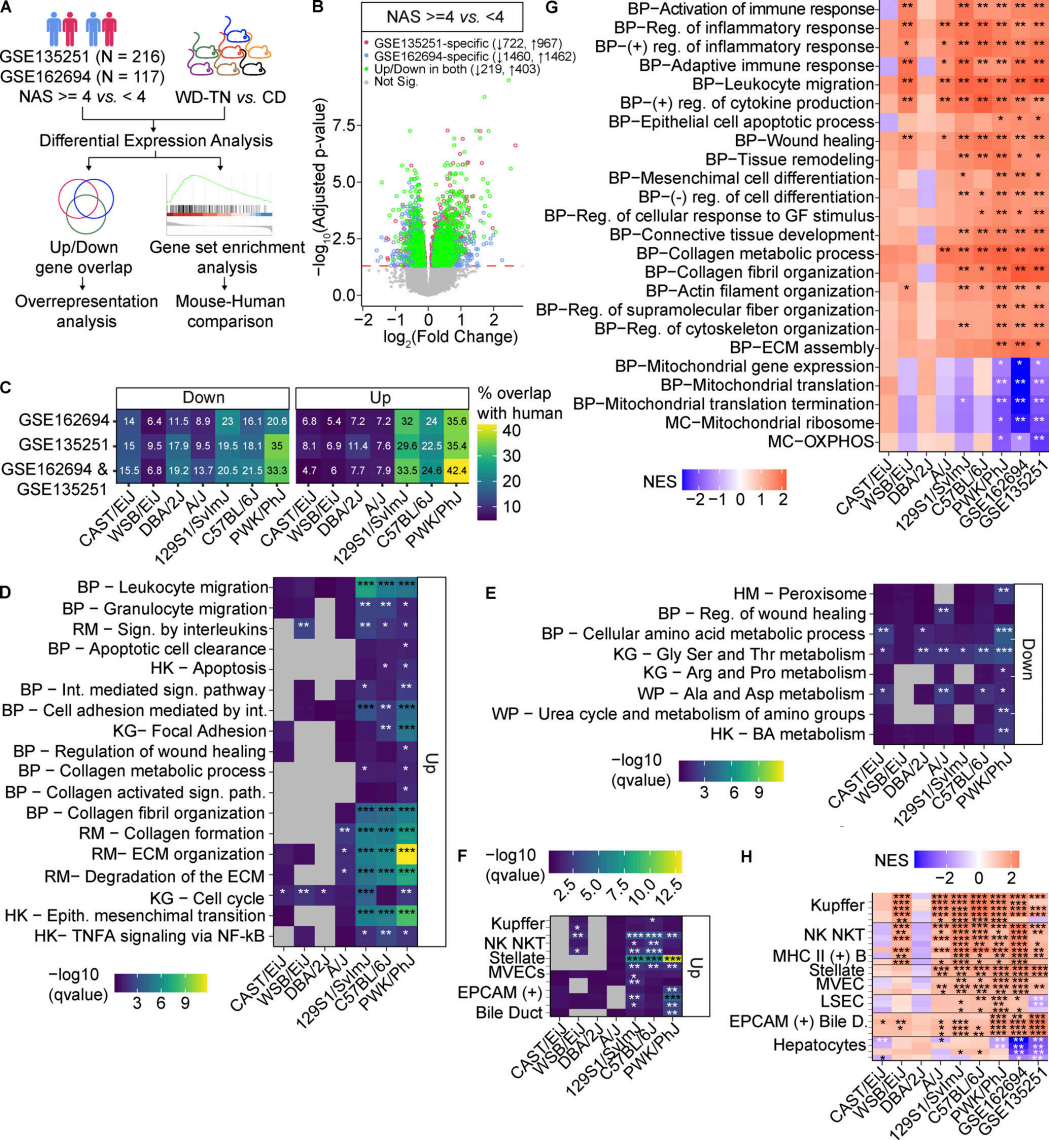
**The PWK/PhJ transcriptional response to WD-TN recapitulates changes seen in human NASH. (A)** Diagram of the bioinformatic pipeline. Differential expression analysis is performed on mouse and human RNA-seq data and transcriptional changes are characterized by logFC-ranked GSEA and by ORA on overlapping significantly DEGs. **(B)** Volcano plot of DEGs (NAS ≥ 4 vs*.* NAS < 4) for the two human liver datasets. Numbers indicate the significant up-/downregulated genes. Red line indicates significance threshold (Benjamini–Hochberg adjusted P value = 0.05); gray indicates non-DEGs; green indicates genes significantly up- or downregulated in both human datasets. **(C)** Direction-specific percentage of human DEGs overlapping in the two human datasets, their overlap, and in each of the seven strains. **(D)** Representative ORA-enriched gene sets for the upregulated gene groups defined in C. Gray indicates missing value. **(E)** Representative ORA-enriched gene sets for the downregulated gene groups defined in C. **(F)** ORA of liver-specific cell type enrichment for the upregulated gene groups defined in C. Every row represents a gene set for a cell type sub-population. **(G)** Representative GSEA-enriched gene sets. **(H)** Cell type GSEA enrichment results. Rows represent gene sets for cell type sub-populations. For all panels: C57BL/6J-CD *n* = 8, C57BL/6J-WD *n* = 8, DBA/2J-CD *n* = 6, DBA/2J-WD *n* = 7, A/J-CD *n* = 6, A/J-WD *n* = 6, 129S1/SvlmJ-CD *n* = 7, 129S1/SvlmJ-WD *n* = 7, WSB/EiJ-CD *n* = 6, WSB/EiJ-WD *n* = 6, CAST/EiJ-CD *n* = 8, CAST/EiJ-WD *n* = 8, PWK/PhJ-CD *n* = 6, PWK/PhJ-WD *n* = 7. Each group of mice was assayed in two independent cohorts. FDR-corrected P values: *, q < 0.05; **, q < 0.01; ***, q <0.001. BP, GO-BP; MC, mitocarta; RM, reactome; HK, Hallmark; KG, KEGG; WP, WikiPathways; reg., regulation; (+), positive; (−), negative; GF, growth gactor; Int., interferon; Sig., signaling; epith., epithelial; Cs, cells; Bile D, bile duct; BA, bile acid, ECM, extracellular matrix.

**Figure S5.**
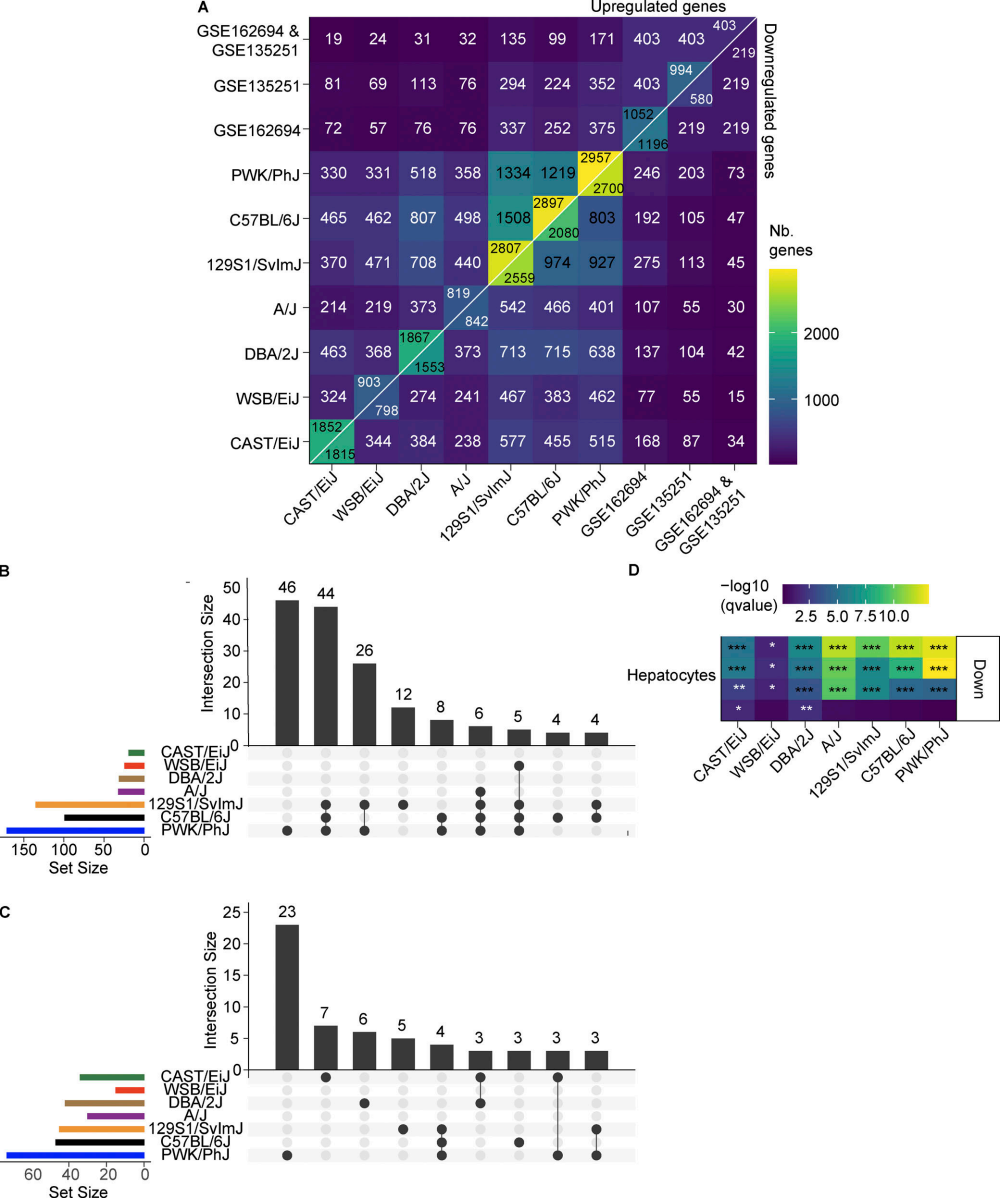
**The PWK/PhJ transcriptional response to WD-TN recapitulates changes seen in human NASH. (A)** Pairwise significantly (alpha = 0.05) DEGs overlap size in human (NAS ≥ 4 vs. <4, concordant in the two datasets) and mouse (WD-TN vs. CD). Upper diagonal: Upregulated genes; lower diagonal: downregulated genes. **(B)** Upset plot showing the exclusive intersection sizes for the overlapping upregulated genes defined in Fig. 8 B. Only the first nine intersections are shown. **(C)** Upset plot showing the exclusive intersection sizes for the overlapping downregulated genes defined in Fig. 8 B. Only the first nine intersections are shown. **(D)** ORA liver–specific cell type enrichment for the downregulated gene groups defined in Fig. 7 C. Every row represents a gene set for a cell type sub-population. FDR-adjusted P values: *, q < 0.05; **, q < 0.01; ***, q < 0.001.

To evaluate whether PWK/PhJ extreme susceptibility to WD-TN is relevant for human disease modeling, we investigated the overlap with human liver signatures associated with increased NAS scores. We selected two human cohorts of 216 and 117 individuals with liver expression and histology assessment and compared subjects with NAS ≥ 4 to NAS < 4 (Fig. 8 A). This allowed us to define a disease signature of 219 and 403 genes that were concordantly and significantly (alpha = 0.05) down and upregulated, respectively, in both human datasets (Fig. 8 B). We then compared this signature with the transcriptional response of different mouse strains to WD-TN (Fig. 8 C and Fig. S5 A). Interestingly, PWK/PhJ emerged as the strain with the largest overlap of upregulated genes (171 genes or 42.4% of the human signature), followed by 129S1/SvlmJ (135 genes or 33.5% of the human signature), and C57BL/6J (99 genes, 24.6% of the human signature). The number of intersecting genes is not simply dictated by the number of DEGs in each strain. PWK/PhJ mice have almost twice the number of upregulated human signature genes yet a similar number of total upregulated genes as C57BL/6J (Fig. S5 A). In addition, PWK/PhJ has 46 unique human signature genes and shares 44 with 129S1/SvlmJ and C57BL/6J (Fig. S5 B). The resistant strains had very little overlap with the human disease signature. Similar overlaps were observed in the downregulated genes (Fig. S5 C).

To functionally characterize the overlap between the mouse response to WD-TN and the human disease signature, we performed ORA on the common genes. Among the upregulated genes, we observed an enrichment of terms related to fibrosis, tissue remodeling, and inflammation (Fig. 8 D). An analogous enrichment analysis on the common downregulated genes showed an enrichment of peroxisome, amino acid, and urea cycle–related pathways (Fig. 8 E). In addition, commonly upregulated genes were enriched for cell type–specific signatures of Kupffer, natural killer (NK), NKT, and hepatic stellate cell subpopulations (Fig. 8 F; see also Materials and methods). While commonly downregulated genes were enriched for cell type–specific signatures of hepatocytes (Fig. S5 D).

We then asked whether the global responses to WD-TN in mice and high vs. low NAS score in humans were qualitatively similar. For that, we performed GSEA on the expression changes in mice and humans (Fig. 8 G). Inflammation, fibrosis, and tissue remodeling are positively enriched in both in humans and in the WD-TN–sensitive strains. Interestingly, mitochondria-related gene sets were downregulated only in human datasets and PWK/PhJ mice. Thus, mitochondria alterations that may drive disease susceptibility in PWK/PhJ mice also represent a key feature of human NASH. Cell type enrichment again pointed to an increase in fibrotic and immunogenic cell types such as hepatic stellate, Kupffer, NK, NKT, and B cells both in humans and in the WD-TN–sensitive strains (Fig. 8 H). Taken together, our results indicate that PWK/PhJ is the strain with the closest transcriptional signature to human NASH and mitochondrial alterations are a common feature of PWK/PhJ mice and human NASH.

### A web resource on inter-strain variation in metabolic disease susceptibility

The phenotypic traits, transcriptome, and proteome data collected in this study can be explored with an online, interactive interface (https://lisp-lms.shinyapps.io/CC_Founders_NASH/). This resource enables researchers to examine the variation induced by WD and TN in each phenotype collected and in each mouse strain, and to choose the appropriate mouse model and the best experimental design for study.

## Discussion

Pre-clinical disease models are essential for drug discovery and drug testing. Many mouse models exist for NAFLD/NASH; however, none of them seem to reproduce the complexity of the human disease. Besides consuming unhealthy diets high in fat and sugar content, humans live almost constantly in their thermoneutral zone, which minimizes energy expenditure (Ganeshan and Chawla 2017). However, laboratory mice are usually housed in mild cold stress conditions (Ganeshan and Chawla 2017). Here, to reproduce experimental conditions closer to human, we used an experimental design that combined WD and TN housing to induce NASH in seven genetically diverse mouse strains. The cardiovascular physiology and metabolism of mice change considerably when they are housed at TN. Mice housed at TN have lower heart rate, lower energy expenditure, and increased metabolic inflammation (Ganeshan and Chawla 2017; Tian et al. 2016). Accordingly, in our study, we observed a strong reduction in energy expenditure when mice were housed at TN compared to RT. All strains housed at TN also had increased plasma LDL-cholesterol, independently of the diet (Fig. 4 C). This is in line with a recent study that found that cold exposure increases cholesterol conversion to bile acids in the liver to promote adaptive thermogenesis (Worthmann et al. 2017). Additionally, mice housed in thermoneutral conditions had a remarkable reduction in lean mass and more severe metabolic outcomes. TN housing was already shown to induce a more severe liver damage in C57BL/6J mice fed a HFD (Giles et al. 2017). However, in this first study, the development of overt fibrosis was not observed. Here, by extending the experiment to seven genetically diverse mouse strains and using a WD, we obtained a full spectrum of phenotypes. The PWK/PhJ strain was the most sensitive to the environmental challenges; PWK/PhJ mice had the highest inflammation score, showed the highest increase in immune cell infiltration, and were the only ones to develop extensive liver fibrosis. Furthermore, PWK/PhJ mice were the most similar to human NASH at the molecular level. Of note, significant liver fibrosis is very difficult to induce in dietary mouse models and normally takes several months to develop (Ipsen et al.,2020; St. Rose et al. 2022). We also identified 129S1/SvlmJ and C57BL/6J strains as highly sensitive to the environmental challenges, with development of liver steatosis and increased inflammation but with a less severe liver phenotype. Conversely, CAST/EiJ mice were found to be completely resistant to the clinical alterations as well as to the hepatic histological alterations induced by WD-TN. This suggests that, although WD-TN is overall more suited than more classical experimental designs (HFD-RT) to induce metabolic alterations and liver disease, the different strains display different degrees of disease severity. These data together suggest that experimental conditions closer to human (different genetic backgrounds and WD combined with TN) may unravel novel and/or more severe phenotypes in the mice that are more similar to the metabolic alterations observed in human and may be more suitable to model diseases and test drug targets (Ganeshan and Chawla 2017). Additionally, the genetic background chosen in metabolic studies is as important as other experimental parameters, such as the type of diet and the housing temperature, and should be carefully considered.

The transcriptional reprogramming of the liver induced by WD-TN is also highly dependent on the genetic background, with a surprisingly large proportion of strain-specific DEGs. Among the strain-specific pathways, mitochondria were particularly dysregulated in PWK/PhJ mice, a phenotype that was not observed in any of the other strains. Upon WD feeding and thermoneutral housing, we indeed observed reduced mRNA, protein expression, assembly and activity of mitochondrial respiratory complexes and supercomplexes, and reduced levels of NAD^+^ and ATP in PWK/PhJ mice, underlining the importance of mitochondrial dysfunction in the progression of NASH. This suggests that PWK/PhJ mitochondria may be more sensitive to damage induced by metabolic challenges and may be the reason why this strain progresses to a more severe liver phenotype. Further investigations are however required to test this hypothesis.

Altogether, our results emphasize that the genetic background of the animal model used is a strong determinant of the extent of phenotypic changes triggered by environmental challenges. Therefore, gene–environment interactions should be taken into account when designing pre-clinical metabolic studies, and care should be used when interpreting results and testing drugs in mice from a single genetic background (Nadeau and Auwerx 2019; Nelson et al. 2022).

In summary, our study dissected gene–environment interactions in the development of NAFLD/NASH and identified in the PWK/PhJ a novel NASH mouse model with features similar to human NASH. Due to these characteristics, the PWK/PhJ strain may become instrumental for the study of NASH pathogenesis and the discovery and testing of novel approaches to manage this widespread disease. Our data are made publicly available and can be easily explored through our online app (https://lisp-lms.shinyapps.io/CC_Founders_NASH/), which will help researchers choose the best experimental design and genetic background for metabolic studies in the mouse.

### Limitations of the study

Although we were able to point out specific regulatory pathways that are changed upon challenge by WD-TN, one limitation of the present study is that it was not designed to identify specific genes that underpin these changes. Mapping causal loci and genes that predispose to susceptibility and/or to protection against the various metabolic traits and NASH will be the subject of a more extensive genetic study, in which a cross between sensitive and resistant strains will be performed. Due to the lack of longitudinal data, we cannot determine whether the differences observed between strains would in the long term be flattened or whether the mitochondrial alterations observed in PWK/PhJ are a cause or a consequence of the disease progression. Further investigation will be required for an in-depth characterization of the disease progression in PWK/PhJ mice. Lastly, whether the different responses of the liver to metabolic challenges that we observe in different strains are cell autonomous or come from other tissues, other systemic effects and/or differences in inter-organ communication remains to be defined and will likewise be the subject of further investigation.

## Materials and methods

### Choice of mouse models

This study used seven domesticated (C57BL/6J, DBA/2J, A/J, 129S1/SvlmJ) or wild-derived (CAST/EiJ, PWK/PhJ, WSB/EiJ) inbred mouse strains drawn from founders of the well-characterized BXD and collaborative cross-panels, which are well known for their genetic diversity. Only male mice were used. The CC founder strains NOD/ShiLtJ and NZO/HlLtJ were excluded because they naturally develop diabetes and other symptoms in the absence of environmental challenges (Kollmus et al. 2020; NOD: diabetes and immune defects; NZO: severe obesity and diabetes).

### Mouse handling

Mouse strains were imported from Charles River and bred at the École Polytechnique Fédérale de Lausanne (EPFL) animal facility for more than two generations before incorporation into the study. Mice were housed with two to five animals per cage under a 12-h light/dark cycle, with ad libitum access to food and water at all times. Starting from 6 wk of age, all mice were housed at 30°C (TN). From 7 wk of age, the mice were fed WD (Research Diets D12079B; 40% kCal from fat, 17% kCal from protein, and 43% kCal from carbohydrates), or a matched CD (Research Diets D16042904B; 10% kCal from fat, 17% kCal from protein, and 73% kCal from carbohydrates). Strains were entered into each group randomly. Body weight was measured weekly from 7 wk of age until sacrifice. In vivo phenotyping tests started after 11 wk of diet (at 18 wk of age) and were performed every 2 wk to reduce the stress on the animals following the pipeline shown in Fig. 1 A. All animal experiments were performed according to Swiss ethical guidelines and approved by the Service de la Consommation et des Affaires Vétérinaires of the Canton de Vaud (license VD3418).

### Tissue collection

Mice were sacrificed at 24 wk of age after 17 wk of diet treatment. Mice were fasted for 4 h in the morning before sacrifice, and they were sacrificed between from 1:30 and 4 p.m. Before sacrifice, mice received isoflurane anesthesia followed by a complete blood draw from the vena cava and perfusion with cold phosphate-buffered saline. Immediately after, liver, kidney, heart, spleen, gastrocnemius, brown adipose tissue, epididymal, and subcutaneous white adipose tissue were collected and flash-frozen in liquid nitrogen. The blood was placed into EDTA-coated tubes and centrifuged at 4,500 revolutions per minute (rpm) for 10 min at 4°C before. The plasma supernatant was collected and flash-frozen in liquid nitrogen for plasma analyses. Parts of the liver and kidney were stored in formalin or optimal cutting temperature compound for histological analysis.

### Body composition analysis (Echo-MRI)

Body composition analysis was performed at 18 wk of age. Each mouse was placed briefly in an Echo-MRI (magnetic resonance imaging) machine (the 3-in-1; Echo Medical Systems), where lean and fat mass are recorded, along with total body weight, taking ∼1 min per individual.

### Indirect calorimetry

At 18 wk of age, after 11 wk of diet treatment, the mice were housed in the Comprehensive Lab Animal Monitoring System (CLAMS; Columbus Instruments) for 48 h. Mice were housed in individual metabolic cages and movement, and oxygen consumption (VO_2_), and carbon dioxide production (VCO_2_) were measured every 16 min. The first 24 h were considered adaptation, and the second 24 h were used for data analysis.

### Oral glucose tolerance test (OGTT)

For the OGTT, mice were fasted overnight and on the morning of the experiment received a gavage of a 20% glucose solution in water (10 ml [2 g]/kg body weight). Blood glucose levels were measured from the tail vein using a glucometer before the gavage and 15, 30, 45, 60, 90, 120, 150, and 180 min after. Blood was also collected at 0 (pregavage), 15, and 30 min to measure fasting insulin and glucose-stimulated insulin secretion.

### Intra-peritoneal insulin tolerance test (ipITT)

For the ipITT, mice were fasted for 4 h in the morning. The test was performed in the afternoon. Insulin (0.5 U/kg body weight) was injected intraperitoneally. Blood glucose levels were measured from the tail vein using a glucometer prior to injection and after 15, 30, 45, 60, 90, and 120 min after injection.

### Plasma analyses

Plasma parameters were measured on two-times diluted samples (1:1 ratio of plasma to diluent) using DimensionXpand Plus (Siemens Healthcare Diagnostics). The biochemical tests were performed according to the manufacturer kit for each parameter: enzymatic creatinine (DF270B; Siemens Healthcare), glucose (DF40; Siemens Healthcare), high-density lipoprotein (DF48B; Siemens Healthcare), LDL (DF131; Siemens Healthcare), cholesterol (DF27; Siemens Healthcare), transaminase ASAT (DF41A; Siemens Healthcare), transaminase ALAT (DF143; Siemens Healthcare), urea nitrogen (DF21; Siemens Healthcare), and triglycerides (DF69A; Siemens Healthcare).

Plasma levels of TIMP-1, FGF-21, and GDF-15 were measured using the Mouse Premixed Multi-Analyte Kit (LXSAMSM; R&D Systems) in a Luminex 200 system following the manufacturer’s instructions.

### BN-PAGE and in-gel activity

The BN-PAGE and in-gel activity assay protocols were described in detail previously (Jha et al., 2016). Briefly, 15 mg of frozen liver tissue were homogenized in ice-cold isolation buffer (0.2 M sucrose, 10 mM Tris, 1 mM EGTA/Tris pH 7.4, pH adjusted to 7.4 with 1 M HEPES buffer, protease inhibitors). Following centrifugation, isolated mitochondria protein content was quantified using detergent-compatible protein assay (Bio-Rad). For BN-PAGE immunoblotting and in-gel activity, 50 mg mitochondria extract was solubilized using 5% digitonin. Electrophoresis of solubilized mitochondrial proteins was performed using the NativePAGE system (Novex) using 3–12% gradient gels. For immunoblotting, samples were run at 150 V for 30 min and at 250 V for an additional 90 min. Proteins were transferred on a polyvinylidene fluoride membrane using an iBlot Gel Transfer device (Invitrogen) and incubated with primary antibodies (Anti-OXPHOS Complex Kit [cat. no. ab110413; Abcam] and Anti-MTCO1 antibody [ab14705; Abcam]) to detect total OXPHOS proteins. Immunostaining of the membrane was performed using Western Breeze Chromogenic Immunodetection System (Invitrogen). For complex I and IV in-gel activity assays, samples were run at 150 V for 30 min and at 250 V for additional 150 min to obtain maximal separation of supercomplex bands. Gels were run at 4°C to preserve enzymatic activity. After electrophoresis, gels were incubated first with complex IV substrate solution (Jha et al., 2016) until the appearance of brown bands indicative of complex IV activity. The gels were subsequently incubated in complex I substrate solution (Jha et al., 2016) at 21°C (RT) until the appearance of purple bands indicative of complex I activity.

### Western blot

For Western blot, 5 mg of radioimmunoprecipitation assay (RIPA) buffer–solubilized mitochondria were mixed to 4× Laemmli buffer and loaded on a NuPAGE 4–12% Bis-Tris Gel (NP0336BOX; Thermo Fisher Scientific). Gels were run at 200 V for 1 h in 3-(*N*-morpholino)propanesulfonic acid–SDS running buffer. Proteins were then transferred to polyvinylidene fluoride membranes at 100 V for 2 h on ice. Membranes were blocked with 5% BSA in a mixture of Tris-buffered saline and Tween 20 for 1 h at 21°C and were then incubated overnight at 4°C with total OXPHOS rodent WB antibody cocktail (#ab110413, 1:1,000; Abcam). After three washes with a mixture of Tris-buffered saline and Tween 20, membranes were incubated with HRP-conjugated anti-mouse secondary antibody (1:2,000). Images were quantified by densitometry using Fiji software and normalized to ponceau staining.

### ATP quantification

For ATP measurement, 15–20 mg of frozen liver sample was homogenized in RIPA buffer with protease inhibitors. Homogenized samples were rotated for 20 min at 4°C and then spun down at 11,000 rpm for 20 min at 4°C. The supernatant was used for protein quantification with detergent-compatible protein assay (Bio-Rad). Protein samples were diluted to 1 mg/ml with RIPA buffer. ATP concentration was measured using CellTiter-Glo Luminescent Cell Viability Assay (G755A; Promega). Protein samples (20 ml of 20 mg) were assayed per well; the volume in each well was brought to 100 ml with PBS and then mixed with 100 ml CellTiter-Glo reagent. After 10 min incubation at 21°C, the luminescence signal was recorded.

### mtDNA/nucDNA ratio

mtDNA abundance was quantified as described (Quiros et al. 2017), with some modifications. In short, DNA was extracted from 15 mg of frozen liver samples using the NucleoSpin Tissue kit (#740952; Macherey-Nagel) following the manufacturer’s instructions. The resulting genomic DNA was diluted to 10 ng/ml and 2 ml (20 ng) were used for quantitative PCR on a Roche LightCycler 480 using TB Green Premix Ex Taq (RR420W; Takara) mastermix. For mtDNA quantification, primers recognizing three different mitochondrial genes were used: 16S rRNA (forward: 5′-CCG​CAA​GGG​AAA​GAT​GAA​AGA​C-3′, reverse: 5′-TCG​TTT​GGT​TTC​GGG​GTT​TC-3′), ND1 (forward: 5′-CTA​GCA​GAA​ACA​AAC​CGG​GC-3′, reverse: 5′-CCG​GCT​GCG​TAT​TCT​ACG​TT-3′), dloop (forward: 5′-AAT​CTA​CCA​TCC​TCC​GTG​AAA​CC-3′, reverse: 5′-TCA​GTT​TAG​CTA​CCC​CCA​AGT​TTA​A-3′). For nucDNA quantification, primers against the Hk2 gene were used (forward: 5′-GCC​AGC​CTC​TCC​TGA​TTT​TAG​TGT-3′, reverse: 5′-GGG​AAC​ACA​AAA​GAC​CTC​TTC​TGG-3′).

### Liver NAD^+^ measurement

NAD^+^ was extracted using acidic extraction method and analyzed by high-performance liquid chromatography (HPLC) mass spectrometry as described (Yang and Sauve 2006). Briefly, ∼10 mg of frozen ground livers were used for NAD^+^ extraction in 10% perchloric acid and neutralized in 3 M K_2_CO_3_ on ice. After final centrifugation, the supernatant was filtered and the internal standard (NAD^+^-C13) was added and loaded onto a column (150 Å∼2.1 mm; Kinetex EVO C18, 100 Å). HPLC was run for 1 min at a flow rate of 300 ml/min with 100% buffer A (methanol/H_2_O, 80/20% vol/vol). Then, a linear gradient to 100% buffer B (H_2_O + 5 mM ammonium acetate) was performed (at 1–6 min). Buffer B (100%) was maintained for 3 min (at 6–9 min), and then a linear gradient back to 100% buffer A (at 9–13 min) started. Buffer A was then maintained at 100% until the end (at 13–18 min). NAD^+^ eluted as a sharp peak at 3.3 min and was quantified on the basis of the peak area ratio between NAD^+^ and the internal standard and normalized to tissue weight and protein content.

### Citrate synthase activity

Citrate synthase activity was measured following the protocol of Sigma’s citrate synthase assay kit (CS0720; Sigma-Aldrich).

### Liver triglycerides and cholesterol measurement

For liver triglycerides and cholesterol measurements, lipids were extracted as described previously (Jha et al. 2014). Triglyceride and cholesterol content in hepatic lipid fraction was quantified with enzymatic assays (Roche) using glycerol and cholesterol as standards.

### RNA isolation

For mRNA, livers were crushed in liquid nitrogen, and then 10 mg of tissues were suspended in TRIzol (Invitrogen) and homogenized with stainless steel beads using a TissueLyser II (Qiagen) at 30 Hz for 2 min. RNA was extracted and purified using Direct-zol-96 RNA kits (Zymo Research). mRNA concentration was measured for all samples. All samples passed a quality check of purity (NanoDrop) and fragmentation (FragmentAnalyzer).

### RNA-seq

RNA libraries were prepared for sequencing using SMARTER mRNA-Seq Library Prep Kit standard protocols. RNA-seq was performed on a BGISEQ-500. FastQC (default parameters) was used to verify the quality of the mapping. No low-quality reads were present, and no trimming was needed. The STAR aligner was used for mapping the RNA-seq data to the C57BL/6J reference genome and determining gene counts. We did not use distinct genomes for each strain due to various genome quality differences between mouse strains that could create bigger artifacts than mapping all strains on the same reference genome in terms of mapping efficiency and gene count estimation. Differential expression was performed using Limma-Voom with package version 3.42.2 (Ritchie et al. 2015) on trimmed mean of M values (TMM)–normalized counts computed with EdgeR calcNormFactors (Robinson et al., 2010). DEGs were determined with the contrast WD vs. CD. The significance threshold was set at 5% after Benjamini–Hochberg multiple testing correction.

### Proteomics analysis

#### Sample preparation

400 µl of guanidine hydrochloride solution in Tris-buffered saline at pH 8 was added to ∼20 mg liver frozen powder. Samples were homogenized in the Tissue Lyser II (Qiagen) for 2 min at 25 Hz followed by centrifugation for 10 mins. at 14,000 rpm at 4°C. The supernatants were heated up to 65°C for 5 min, and bath sonicated at medium strength for 5 min. The samples were subsequently centrifuged at 4,000 rpm for 10 min at 4°C, and the supernatant was collected. Protein concentrations were determined with a Bradford assay, and 25 µg of protein was used for the proteomics sample preparation procedure. The samples were then reduced, alkylated, and trypsinized. After trypsinization, peptides were cleaned using SOLAμ solid phase extraction plates and eluted in an 80% acetonitrile solution with 0.1% trifluoroacetic acid water. Peptides were then dried and analyzed by liquid chromatography with tandem mass spectrometry (LC-MS/MS).

#### BoxCar Proteomics HPLC-MS analysis

A library was first created by mixing equal amounts of proteins from each sample. Digested and desalted samples were fractionated into 24 fractions using an OFF-gel PI-based system (3100; Agilent) as described by the manufacturer. Each fraction was desalted using SDB-RPS Stage Tips and dried by vacuum concentrator. The resulting 24 samples were resuspended in 2% acetonitrile; 0.1% formic acid and 1 µg was injected for LC-MS/MS analysis over 90 min. gradients using standard shotgun data-dependent acquisition mode. Individual samples were acquired using a BoxCar LC-MS/MS method described elsewhere (Meier et al. 2018). Briefly, due to protein high dynamic range of the samples, BoxCar termed acquisitions were performed through sequential and interspaced narrow m/z windows ultimately covering the full mass range. A first full scan was acquired followed by two BoxCar-based ones covering 400 to 1200 m/z range. The nano-flow separations were performed on an Ultimate 3000 RSLC nano-UPLC system (Thermo Fisher Scientific) connected online with an Exploris 480 Orbitrap mass spectrometer (Thermo Fisher Scientific) at the EPFL Proteomics Core Facility. A capillary precolumn (Acclaim PepMap C18; 3 μm-100 Å; 2 cm × 75 μm ID; Thermo Fisher Scientific) was used for sample trapping and cleaning. Analytical separations were performed at 250 nl/min over a 90-min biphasic gradient on a 50-cm long in-house packed capillary column (75 μm ID; ReproSil-Pur C18-AQ; 1.9 μm silica beads; Dr. Maisch). Initial full scans were acquired with a resolution of 120,000 (i.e., at 200 m/z) as well as the following two BoxCar scans. The five most intense parent ions were selected from the first full scan and fragmented by high-energy collision dissociation with a normalized collision energy of 30%, using an isolation window of 1.4 m/z. Fragmented ion scans were acquired with a resolution of 15,000 (i.e., at 200 m/z) and selected ions were then excluded for the following 25 s.

#### Data analysis

We compiled an in silico *M. musculus* proteome for the seven strains by including all annotated proteins from C57BL/6J, DBA/2J, 129S1/SvlmJ, A/J, CAST/EiJ, PWK/PhJ, and WSB/EiJ in Ensembl release 107 (available at https://www.ensembl.org/info/data/ftp/index.html). The resulting FASTA files were then preprocessed with an in-house script to remove all initiator methionines and signal peptides, as annotated in Uniprot. Thermo raw files were searched with MaxQuant version 2.1.0.0 (Cox and Mann, 2008). Cysteine carbamidomethylation was included as a fixed modification, while methionine oxidation and protein N-terminal acetylation were included as variable modifications. We allowed for a maximum of two missed cleavages and searched the data with MaxQuant’s match-between runs feature, while also enabling the identification of second peptides. Peptide intensities were imported from MaxQuant’s peptides.txt file into R version 4.1.0, running on RStudio Workbench version 1.4.1717-3. Contaminants, reverse sequences, and proteins only identified by modified peptides were removed from the data. Raw intensities were then log_2_-transformed and normalized with robust linear regression normalization, as implemented in the NormalizerDE R package. We then removed proteins identified by a single peptide, as well as peptides with fewer than two identifications. Differential abundance analysis was performed with MSqRob (Goeminne et al., 2016, Goeminne et al., 2018), and included a fixed intercept, as well as fixed effects for strain, diet, and their interaction. The fixed effects for diet, strain, and diet:strain interaction were assigned a combined ridge penalty estimated by exploiting the link between ridge regression and mixed models. We further included random effects for sample and peptide sequence. P values were adjusted for multiple testing with the Benjamini–Hochberg false discovery rate (FDR) procedure.

### Human NASH RNA-seq datasets

#### Differential expression (human)

Public human liver bulk RNA-seq processed counts were downloaded from the Gene Expression Omnibus (GEO) under the accession numbers GSE135251 (Govaere et al. 2020) and GSE162694 (Pantano et al. 2021). Samples (subjects) were grouped according to the NAS stage, resulting, respectively, in 148, 53 samples with NAS ≥ 4 and 68, 64 samples with NAS < 4. Lowly expressed genes were filtered with the edgeR filterByExpr function version 3.28.1 (Robinson et al., 2010). Differential expression was performed using Limma-Voom with package version 3.42.2 (Ritchie et al. 2015) on TMM-normalized counts computed with EdgeR calcNormFactors (Robinson et al., 2010). We determined differential expressed genes with the contrast NAS ≥ 4 vs. NAS < 4 accounted for sex using the following design formula: ∼NAS_group + sex. We set the significance threshold at 5% after Benjamini–Hochberg multiple testing correction.

#### Human–mouse comparison

After differential expression analysis, we performed GSEA analysis using ClusterProfiler version 3.14.3 (Yu et al. 2012) and the Gene Ontology–Biological Process (GO-BP), KEGG, Reactome, Wikipathways, and Hallmark annotations retrieved from MSigDB with the msigdbr package version 7.4.1 (Dolgalev 2021). We also retrieved and used mitochondrial gene sets from Mitocarta version 3.0 (Rath et al. 2021). We performed ORA on the overlapping genes significantly differentially expressed in mouse and human data using the same gene set annotations and ClusterProfiler (Yu et al. 2012), with 10,000 permutations. Multiple testing correction was performed with Benjamini–Hochberg.

#### Estimation of cell type proportions

To estimate the cellular composition of the mouse liver samples, we performed single-cell deconvolution using MuSiC version 0.2.0 (Wang et al. 2019) on raw bulk RNA-seq counts using the default parameters and a maximum number of iteration equal to 1,500. We retrieved liver FACS single-cell RNA-seq processed counts and samples annotations from GEO under accession no. GSE109774 and from the supplementary data provided by the Tabula Muris consortium authors (Schaum et al. 2018). We determined the percentage of immune cells by summing the contributions of NK, Kupffer, and B cells. Endothelial cells annotation refers to endothelial cells of the hepatic sinusoid. A *t* test and Benjamini–Hochberg multiple testing correction was used to test the significance of cell type composition induced by WD-TN compared to CD.

### Liver histology

Liver specimens were collected from all mice immediately after euthanasia and were fixed in 10% neutral buffered formalin for 24–48 h. After fixation, representative specimens from every liver were trimmed and processed with conventional paraffin embedding technique. Paraffin-embedded specimens were then blocked and sliced using rotary microtome at 5-micron thickness. Sliced sections were stained either with H&E or Picro-Sirius red (PSR) using an internal protocol.

### Histopathological analysis

Specimens were examined unbiased for the presence of histopathologic lesions. The NAS was evaluated as previously described (Hübscher 2006; Kleiner et al. 2005). The scoring was performed as follows: (A) Steatosis (amount of lipid vacuoles accumulation): Grade 0 = <5%; 1 = 5–33%; 2 = 33–66%; 3 = >66%, with 0.5 intervals. (B) Hepatocellular ballooning (presence of ballooned cells): 0 = none; 1 = few balloon cells; 2 = many cells/prominent ballooning, with 0.5 intervals. (C) Lobular inflammation: 0 = none; 1 = <2 foci per 200×; 2 = 2–4 foci; 3 = >4 average foci/200× field, with 0.5 intervals. (D) NAFLD: Sum of scores in parameters A, B, and C. (E) Other lesions: Lesions other than those mentioned above were scored on a semi-quantitative score from 0 to 5 (0 = no lesions, 1 = subtle, 2 = mild, 3 = moderate, 4 = severe, 5 = marked).

### Quantitative liver tissue section image analysis for vacuoles and collagen content

Automated tissue section–based quantification of vacuoles in hepatocytes (steatosis) and PSR histochemical staining (surrogate for collagen) was performed using image analysis algorithms in Visiopharm (version 2020.08.0.8126; Visiopharm). Whole-tissue sections (two liver lobes per animal) were defined as regions of interest and included in the analysis. An image analysis algorithm first was applied to segment and quantify areas of tissue comprised of intracytoplasmic lipid vacuoles in hepatocytes. A second algorithm was applied to quantify the PSR staining in the region of interest, excluding the image areas comprised of lipid vacuoles. PSR-positive area was then quantified and expressed as a percent of the total tissue area of interest (excluding image area of lipid vacuoles).

### Quantification and statistical analysis

No statistical methods were used to predetermine sample size. The exact value of *n*, the statistical methods used to determine significance and error bars are described in the figure legends. All replicates represent biological replicates. Statistical tests were performed using R. All P values <0.05 were considered significant; *, P < 0.05; **, P < 0.01; ***, P < 0.001; ****, P < 0.0001.

### Material availability

This study did not generate new unique reagents*.*

### Online supplemental material

Fig. S1 shows that the mouse genetic background is a major determinant of the physiological responses to metabolic challenges (related to Fig. 1 and Fig. 2). Fig. S2 shows that PWK/PhJ mice are the most sensitive to liver damage and NASH progression to fibrosis (related to Fig. 3). Fig. S3 shows a comparison of the effect of HFD-RT and WD-TN on different phenotypes and strain-specific ORA (related to Fig. 4 and Fig. 5). Fig. S4 shows that PWK/PhJ mice have severe mitochondrial dysfunction on WD-TN (related to Fig. 7). Fig. S5 shows that the PWK/PhJ transcriptional response to WD-TN recapitulates changes seen in human NASH (related to Fig. 8). Table S1 lists phenotypes description and abbreviations.

## Supplementary Material

Table S1 shows phenotype descriptions and abbreviations.

SourceData F7 contains original blots for Fig. 7.

SourceData FS4 contains original blots for Fig. S4.

## Data Availability

All the raw data used in this study were deposited at Mendeley Data (https://doi.org/10.17632/dntgsyznzs.1; Benegiamo, 2023). All RNA-seq data were deposited in the GEO database under accession number GSE201819. All the results generated by the present study are available through our online app (https://lisp-lms.shinyapps.io/CC_Founders_NASH/). Any additional information required to reanalyze the data reported in this paper is available from the corresponding authors upon request. Essential scripts used in this study were deposited at Mendeley Data (https://doi.org/10.17632/dntgsyznzs.1; Benegiamo, 2023).
