# Supplementary figures and images for: The genetic background shapes the susceptibility to mitochondrial dysfunction and NASH progression
Source: J Exp Med. 2023 Feb 14;220(4):e20221738. doi: 10.1084/jem.20221738 (PMC9960245; doi:10.1084/jem.20221738)

Figure 7 - Source Data

A

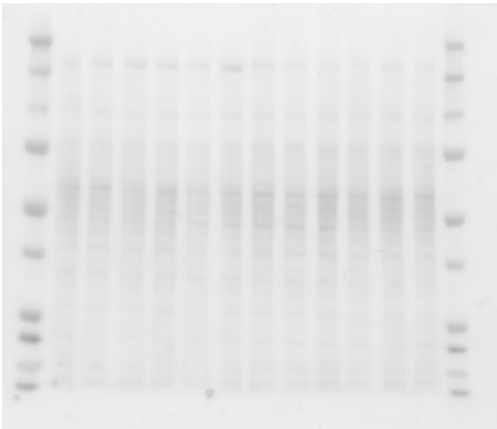

B

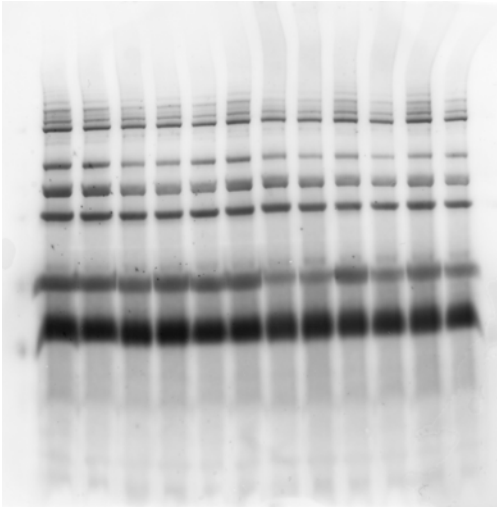

C

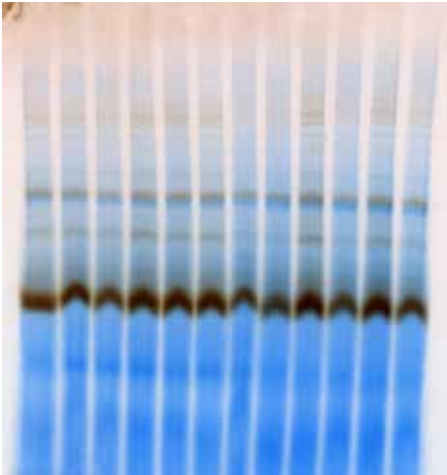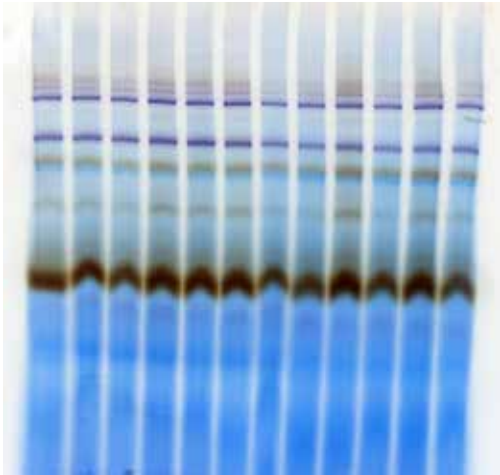

Supplement: SourceData F7 — contains original blots for Fig. 7. [file JEM_20221738_SourceDataF7.pdf]

Figure S4 - Source Data

A

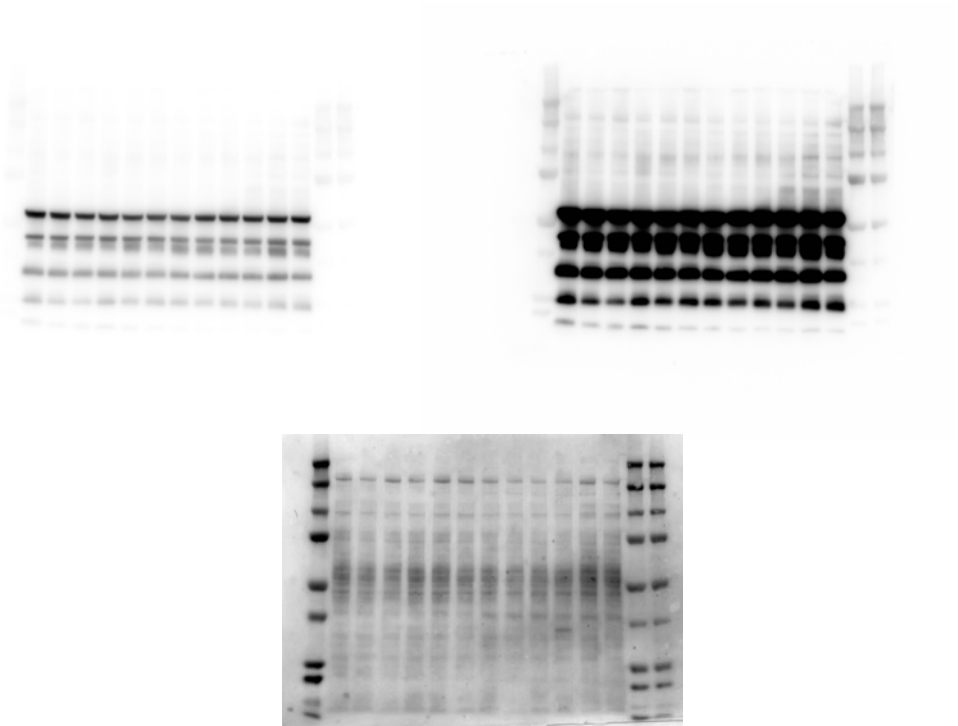

B

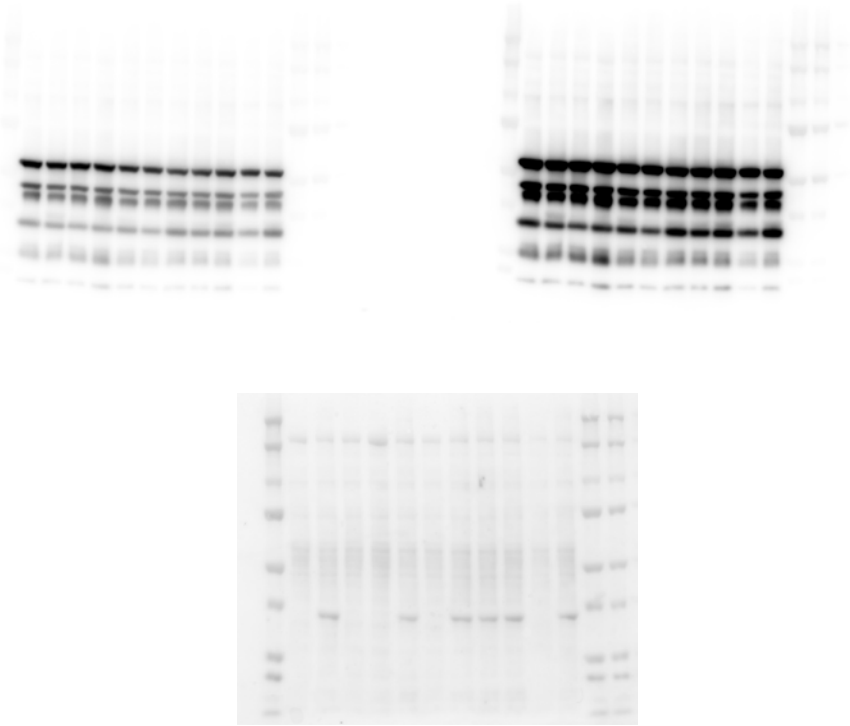

**Figure S4 - Source Data**

**C**

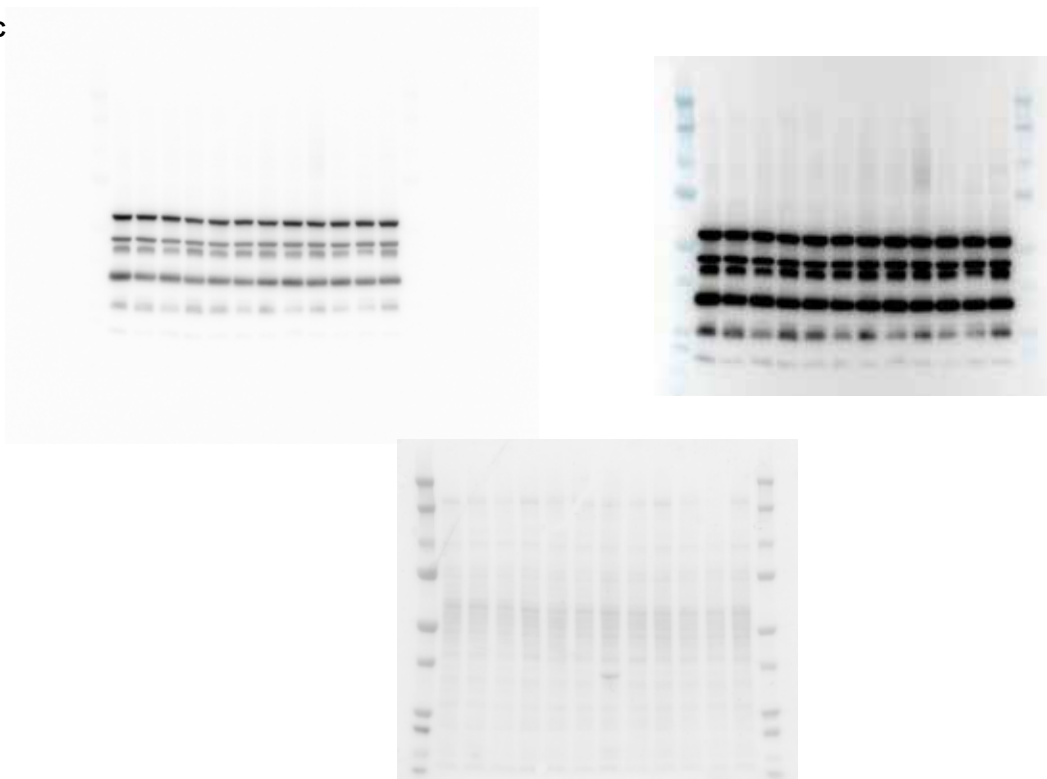

**D**

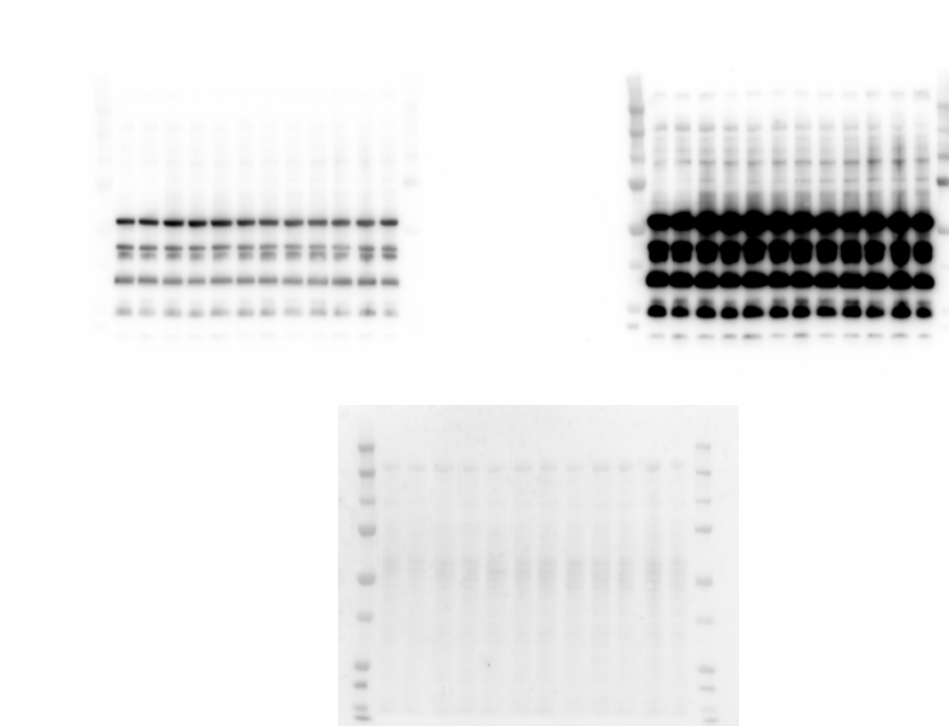

Figure S4 - Source Data

E

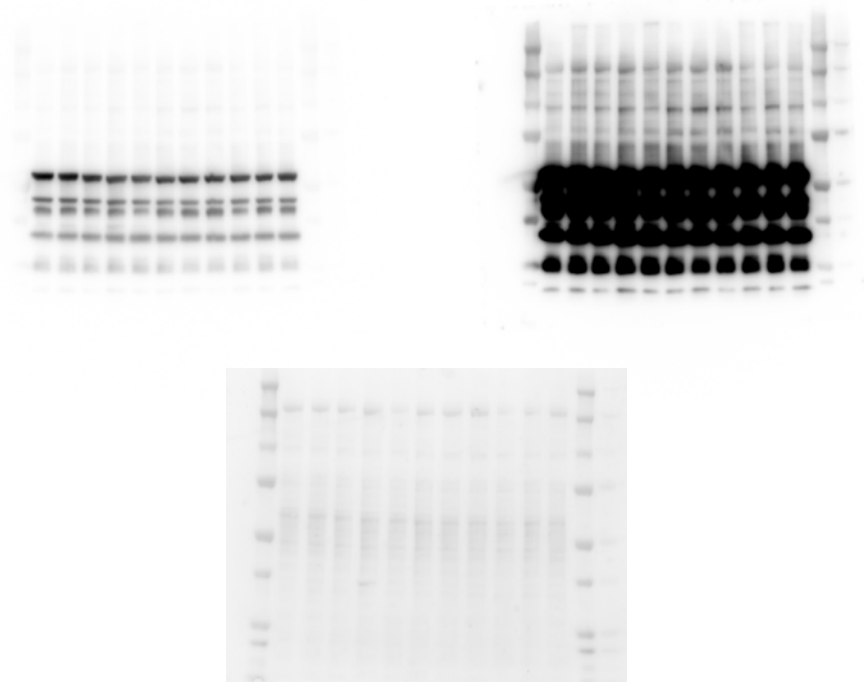

F

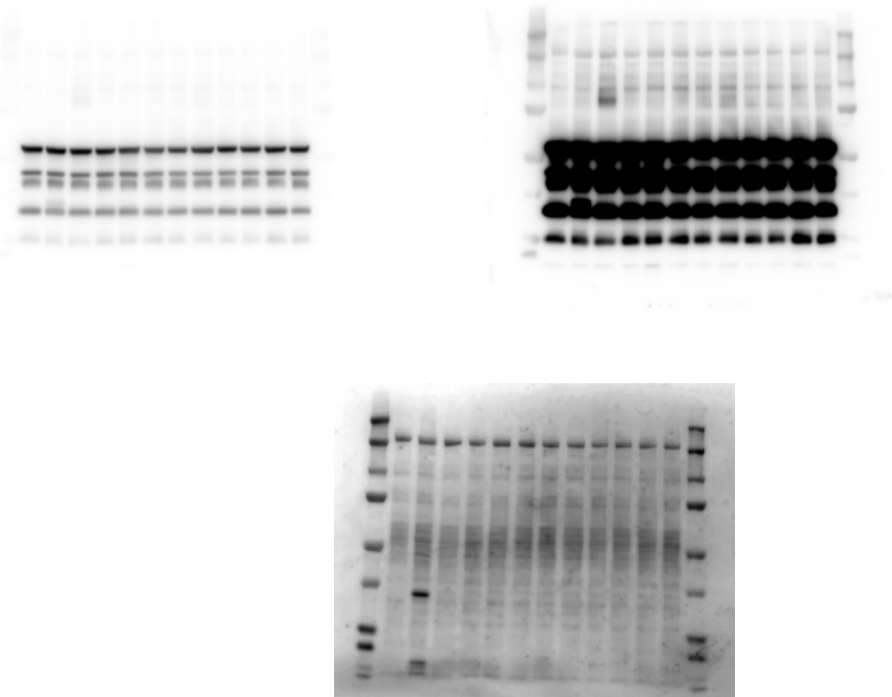

Supplement: SourceData FS4 — contains original blots for Fig. S4. [file JEM_20221738_SourceDataFS4.pdf]
